# Endoscopic Advances in the Diagnosis and Management of Gastroesophageal Reflux Disease

**DOI:** 10.3390/medicina60071120

**Published:** 2024-07-11

**Authors:** Priyadarshini Loganathan, Mahesh Gajendran, Abhilash Perisetti, Hemant Goyal, Rupinder Mann, Randy Wright, Shreyas Saligram, Nirav Thosani, Chandraprakash Umapathy

**Affiliations:** 1 Division of Gastroenterology & Nutrition, The University of Texas Health Science Center San Antonio, San Antonio, TX 78229, USA; drdarshini88@gmail.com (P.L.); gajendran@uthscsa.edu (M.G.); wrightrp@uthscsa.edu (R.W.); 2 Department of Gastroenterology and Hepatology, Kansas City VA Medical Center, Kansas City, MO 64128, USA; abhilash.perisetti@gmail.com; 3 Department of Gastroenterology, Borland Groover, Baptist Medical Center-Downtown, Jacksonville, FL 32207, USA; 4 Department of Gastroenterology, University of Arkansas for Medical Sciences, Little Rock, AR 72205, USA; rupindrmann@yahoo.com; 5 Department of Gastroenterology, Robert Wood Johnson University Hospital, New Brunswick, NJ 08901, USA; drsaligram@yahoo.com; 6 Department of Surgery, McGovern Medical School at UT Health, Houston, TX 77030, USA; nirav.thosani@uth.tmc.edu; 7 Division of Gastroenterology & Nutrition, Audie L. Murphy VA Hospital, San Antonio, TX 78229, USA; umapathy@uthscsa.edu

**Keywords:** GERD, gastroesophageal reflux disease, endoscopy, Narrow Band Imaging, I-scan, FICE, esophageal capsule endoscopy, transoral incisionless fundoplication

## Abstract

Gastroesophageal reflux disease (GERD) is one of the most common diseases that occurs secondary to failure of the antireflux barrier system, resulting in the frequent and abnormal reflux of gastric contents to the esophagus. GERD is diagnosed in routine clinical practice based on the classic symptoms of heartburn and regurgitation. However, a subset of patients with atypical symptoms can pose challenges in diagnosing GERD. An esophagogastroduodenoscopy (EGD) is the most common initial diagnostic test used in the assessment for GERD, although half of these patients will not have any positive endoscopic findings suggestive of GERD. The advanced endoscopic techniques have improved the diagnostic yield of GERD diagnosis and its complications, such as Barrett’s esophagus and early esophageal adenocarcinoma. These newer endoscopic tools can better detect subtle irregularities in the mucosa and vascular structures. The management options for GERD include lifestyle modifications, pharmacological therapy, and endoscopic and surgical interventions. The latest addition to the armamentarium is the minimally invasive endoscopic interventions in carefully selected patients, including the electrical stimulation of the LES, Antireflux mucosectomy, Radiofrequency therapy, Transoral Incisionless Fundoplication, Endoscopic Full-Thickness plication (GERDx™), and suturing devices. With the emergence of these advanced endoscopic techniques, it is crucial to understand their selection criteria, advantages, and disadvantages.

## 1. Introduction

Gastroesophageal reflux disease (GERD) is one of the most common diseases that occurs secondary to failure of the antirefluxflux barrier system, resulting in the frequent and abnormal reflux of gastric contents to the esophagus. The classic symptoms of GERD are heartburn and regurgitation. The extra-esophageal manifestations of GERD include chest pain, cough, ear, nose, or throat symptoms [1,2]. The actual prevalence of GERD is difficult to measure due to various factors. Many individuals with GERD are asymptomatic, including those with Barrett’s esophagus (BE). Furthermore, assessing GERD based on objective testing such as endoscopy and pH testing in population-based studies is impractical [3]. In a systematic review of 102 population-based studies, the prevalence of GERD was found to be 13.3% (95% Confidence interval CI 12.0% to 14.6%) [4,5]. However, significant heterogeneity was found in these studies, with Central American studies reporting the highest GERD prevalence of 19.6% and Asian studies reporting the lowest prevalence rates of 10.0%, particularly in countries of Southeast Asia (7.4%) [5]. Another systematic review of 31 population-based studies reported that the prevalence of GERD in North America ranged between 18.1 and 27.8%, with an increase in prevalence between 1995 and 2005 [1]. In a study utilizing an administrative database, 500,000 patients (about half the population of Montana) were hospitalized with a primary GERD-related diagnosis, and 14.5 million patients (about twice the population of New Jersey) were hospitalized with GERD-related conditions as a secondary diagnosis [6].

The risk factors for GERD can be broadly categorized into demographic and environmental risk factors. In terms of demographic risk factors, gender and race do not seem to influence the prevalence of GERD symptoms in the US, whereas increasing age > 50 years is associated with an increase in GERD symptoms [5,6,7]. Male gender, advancing age, and the white race are associated with a higher risk for erosive esophagitis, BE, and esophageal adenocarcinoma [5,6,7]. The two major environmental risk factors contributing to the rising prevalence of GERD are obesity and helicobacter pylori (HP) gastritis. Central adiposity has been associated with an increased risk of GERD symptoms, erosive esophagitis, BE, and esophageal adenocarcinoma [8]. Obesity increases intragastric pressure and the cytokines produced by the visceral fat, decreasing the lower esophageal sphincter (LES) pressure [8]. The decreasing prevalence of HP infection is also associated with increased GERD and its related complications. This inverse relationship could be related to HP-related gastric atrophy [9,10,11]. Other environmental factors reported to be associated with GERD include tobacco and alcohol, but the associations are relatively weak and unpredictable across different populations. Previous case-control studies have shown family clustering of GERD, especially with twins in 30–45%, suggesting genetic susceptibility [12,13]. This has been proposed to be related to the decreased LES pressure, dysmotility, and hiatal hernia.

GERD is diagnosed in routine clinical practice with classic symptoms of heartburn and gastric content regurgitation. These symptoms are specific to diagnosing GERD and empirically starting short-term proton pump inhibitor (PPI) therapy. Several tests are available for GERD evaluation, but they are typically used in patients with refractory, atypical, or alarming symptoms such as dysphagia, odynophagia, upper gastrointestinal bleeding, weight loss, and anemia [14,15,16,17]. Esophagogastroduodenoscopy (EGD) is an important test used to evaluate GERD complications like erosive esophagitis, BE, or esophageal cancer [16]. However, subtle mucosal changes from early BE-related dysplasia, early adenocarcinoma, or squamous cell dysplasia can be missed with the standard EGD using white light [18,19]. Hence, there has been a recent development in advanced endoscopic imaging techniques and endoscopic attachment tools to improve diagnostic accuracy. Some of the advanced diagnostic endoscopic techniques that have been introduced include narrow-band imaging (NBI), high-resolution, high-magnification endoscopy, chromoendoscopy, autofluorescence imaging, wireless capsule endoscopy, and confocal laser endomicroscopy (CLE). In patients with GERD, only up to 50% of the patients have erosive esophagitis on standard white-light endoscopy (WLE). In these cases, the next step in testing is intraesophageal pH or impedance-pH monitoring to assess for GERD [20]. Understanding that using advanced imaging is not an alternative or substitute for pH studies is essential. pH studies are crucial to detecting GERD, but advanced endoscopic imaging helps detect GERD complications like BE and dysplasia.

**Figure 1:**
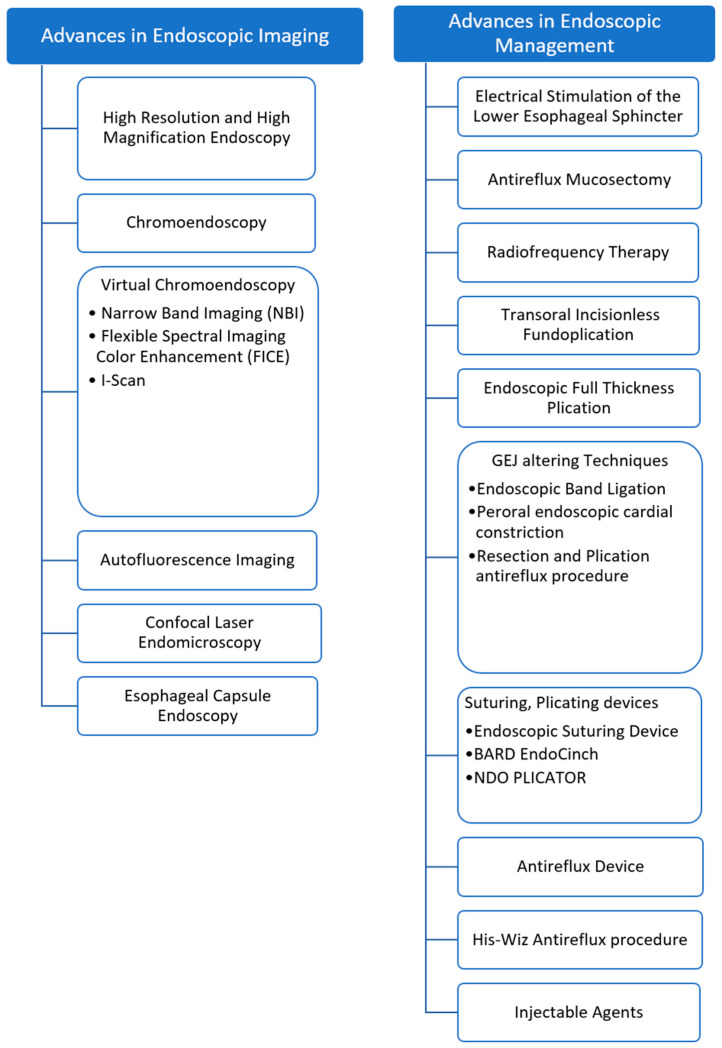
Endoscopic management of GERD.

GERD management typically starts with initiating PPI and complementary lifestyle measures. However, about 20–30% of the patients with erosive esophagitis and about 40% of the patients with non-erosive reflux disease (NERD) may not respond to the PPI treatment [17,21]. Also, some patients who respond well to PPI and need long-term PPI for symptomatic control may prefer not to be on long-term medications. These patients can be potential candidates for surgical or endoscopic intervention to control symptoms and prevent GERD-related complications [22,23]. The endoscopic treatment options include the electrical stimulation of Lantirefluxflux mucosectomy, radiofrequency therapy, transoral incisionless fundoplication, endoscopic full-thickness plication, antirefluxflux techniques utilizing injection devices, and suturing, plicating, or stapling devices [22]. This review has discussed various advanced endoscopic imaging techniques and endoscopic interventions for managing GERD (Figure 1).

## 2. Diagnosis

Diagnosing GERD is often challenging and usually involves evaluating clinical symptoms, assessing the response to acid suppression therapy, and conducting objective tests such as upper endoscopy and esophageal pH monitoring. Patients who present with a history suggestive of uncomplicated GERD, characterized by typical symptoms such as heartburn and/or regurgitation, may be considered for empiric treatment [24]. Typical symptoms that improve with acid suppression provide further indication of pathological esophageal acid exposure, and it is reasonable to consider a diagnosis of GERD. However, patients who do not improve with acid suppression warrant further tests to demonstrate the existence of GERD and evaluate for an alternate diagnosis. Patients presenting with atypical symptoms or non-cardiac chest pain should undergo esophageal function tests before initiating empiric therapy [25]. It is imperative to prioritize endoscopy as the initial diagnostic test for assessing patients who present with dysphagia or other alarm symptoms (such as weight loss or GI bleeding), as well as for those with multiple risk factors for Barrett’s esophagus [16].

**Figure 2:**
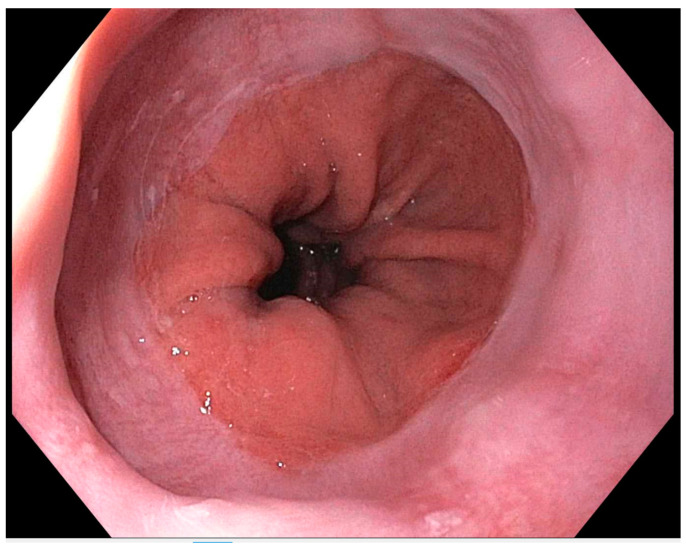
Endoscopic image of the gastroesophageal junction (GEJ) using the high-definition (HD) endoscopy.

When upper endoscopy is unrevealing (Figure 2), ambulatory reflux monitoring performed off PPI therapy can detect an abnormal reflux burden to conclusively diagnose GERD. While both pH monitoring and pH-impedance monitoring accurately assess distal esophageal acid exposure and correlate symptoms with reflux episodes, pH-impedance monitoring offers additional capabilities [26]. It can differentiate between weakly acidic and acidic reflux, evaluate baseline mucosal impedance, and determine the extent of refluxate into the proximal esophagus. Transnasally positioned pH and pH/impedance catheters typically monitor over 24 h, whereas wireless pH telemetry capsule monitoring can extend from 48 to 96 h. The capsule method avoids the discomfort and inconvenience of a transnasal catheter, allowing patients to maintain their daily activities during pH monitoring. Key parameters evaluated during reflux testing include the acid exposure time, reflux event count, and symptom correlation [27]. Impedance pH testing assesses weakly acidic and nonacid reflux, bolus clearance, and the extent of proximal reflux. Reflux symptom association identified through impedance-pH testing may aid in predicting the symptom response to therapy and diagnosing reflux hypersensitivity [28]. In both wireless capsule- and catheter-based reflux tests, the most consistently reliable variables include the total acid exposure time and the composite DeMeester score [29].

In an abnormal pH-impedance test, refractory GERD is diagnosed, the treatment plan should be reevaluated, and management should be optimized with the goal of better reflux control to improve symptoms and, subsequently, improve quality of life and prevent GERD-related complications. When the esophageal evaluation is negative, both esophageal disorders of gut–brain interaction (such as reflux hypersensitivity and functional heartburn) and non-esophageal disorders (rumination, supragastric belching, laryngeal and pulmonary disorders, gastroparesis, and cardiac disease) need to be considered as potential mechanisms of symptom generation [30].

High-resolution manometry (HRM) evaluates motility abnormalities linked with GERD, yet it alone does not confirm GERD diagnosis. Severe GERD often presents with weak lower esophageal sphincter (LES) pressure and ineffective esophageal motility, though no manometric anomaly is specific to GERD. In esophageal impedance-pH monitoring, HRM locates the LES for positioning transnasal pH-impedance catheters. HRM also aids in assessing patients for surgical or endoscopic reflux procedures, particularly to screen for achalasia. Patients with achalasia may exhibit symptoms like heartburn and regurgitation, which can be misdiagnosed as GERD, potentially leading to severe dysphagia post-procedure. Hence, HRM should ideally precede an anti-reflux procedure [31].

Many presentations of GERD manifest as distinct phenotypes with specific predisposing factors and an underlying pathophysiology that diverge from this standard paradigm. Katzka et al. illustrated the major GERD phenotypes with unique pathophysiological and management considerations. Each GERD phenotype has unique features and challenges in diagnosing [32] (Table 1).

## 3. Endoscopic Evaluation for GERD

EGD is not always necessary to evaluate for GERD. Furthermore, the sensitivity of conventional endoscopy for GERD diagnosis is around 40% [33,34]. However, EGD is commonly performed in patients with alarming features or abnormal imaging or for screening for BE in chronic GERD patients with risk factors. Large mucosal breaks (LA grade C or D esophagitis) confirm the diagnosis of GERD. Patients are categorized as having NERD if they have troublesome reflux symptoms but no mucosal breaks on EGD. If >1 cm distal esophageal salmon-colored mucosa is seen, then biopsies are performed to confirm the diagnosis of BE by detecting specialized intestinal metaplasia (IM). In a retrospective study by Eloubeidi et al., the endoscopic diagnosis of BE had a sensitivity of 82% (95% CI 72–92) and a specificity of 81% (95% CI 78–84) [18]. However, the study reported a high negative predictive value (NPV) of 97% and a lower positive predictive value (PPV) of 34%. The length of the involved mucosa was associated with increased odds of a diagnosis of BE (OR = 3.33, 95% CI, 1.54–7.17). Advanced endoscopic imaging techniques, such as virtual chromoendoscopy, have enhanced the identification of Barrett’s metaplasia (Table 2). Currently, using other advanced imaging techniques, such as CLE, to diagnose GERD is not recommended in routine clinical practice.

### 3.1. High-Resolution and High-Magnification Endoscopy

High-resolution and high-magnification endoscopy improves the ability to detect abnormal mucosal changes, especially in cases of minimal change in the esophageal lining. The high-magnification endoscopes have a movable zoom lens at the tip that can magnify 150-fold for improved mucosal examination. In addition, they use 850,000 pixels to provide images with a higher resolution and quality [41]. In a prospective study of NERD patients, the magnification endoscope group identified esophagitis in a significantly higher proportion of patients (64.10%) when compared to the control group (20.5%) [42]. In a retrospective study by Bond et al., dual-focus magnification high-definition endoscopy had increased rates of detecting significant mucosal pathology (OR 1.87, 95% Cl 1.11–3.12) [35]. In a prospective study of 60 patients with suspected BE, high inter-observer variability was observed in detecting IM and dysplasia with enhanced magnification endoscopy (54.9% to 80.7%) [43].

### 3.2. Chromoendoscopy

In chromoendoscopy, a contrast agent is sprayed, which stains the tissue and improves the localization and characterization of the abnormal mucosa [44]. The dyes are categorized into vital and non-vital dyes. Vital dyes such as methylene blue, Lugol’s solution, and toluidine blue are absorbed rapidly by the normal squamous cells. Non-vital dyes, such as crystal violet and indigo carmine, are not absorbed by the cells. They fill the mucosal folds and pits, thereby highlighting the mucosal patterns. Chromoendoscopy is frequently used along with high-resolution and high-magnification endoscopy [41,45,46]. In a study by Yoshikawa et al., using Lugol’s chromoendoscopy in symptomatic GERD patients, 48.7% of patients were found to have positive findings of unstained streaks observed in the distal esophagus when compared with a positive finding of visible esophagitis in 36% of patients on conventional white light EGD [47].

### 3.3. Virtual Chromoendoscopy/Image-Enhanced Endoscopy (IEE)

Virtual chromoendoscopy is an alternative to dye-based chromoendoscopy, which uses digital endoscopic imaging technologies and provides an enhanced visualization of the mucosal surface and blood vessels in real time. The three major technologies that are currently available include Narrow Band Imaging (NBI; Olympus, Tokyo, Japan), Flexible Spectral Imaging Color Enhancement (FICE™) (Fujinon Corp. Tokyo, Japan), and i-scan (Pentax, Tokyo, Japan).

i. **Narrow-Band Imaging (NBI)**

**Figure 3:**
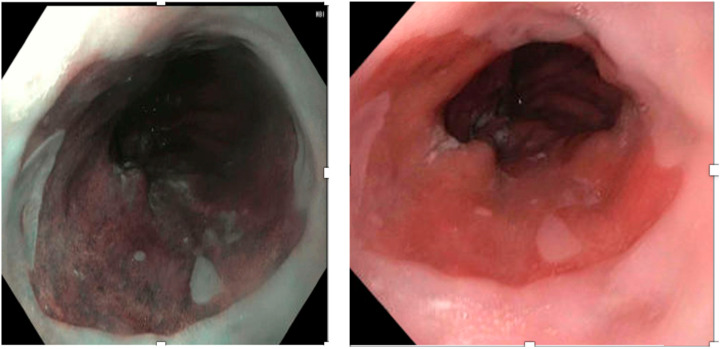
Endoscopic image of the gastroesophageal junction (GEJ) using the Narrow Band Imaging (NBI).

NBI is an innovative optical technology that utilizes a spectral narrow-band filter of 415 + 30 nm to assess surface patterns and the microvascular architecture (Figure 3) [14,45]. It provides a distinct contrast between the esophageal and gastric mucosa since hemoglobin is the primary chromophore in the esophageal tissue in the visible wavelength range, which absorbs blue and green light [46,48]. Combined with high-resolution and high-magnification endoscopy, NBI enables the visualization of the mucosal intra-epithelial papillary capillary loop (IPCL) and submucosal branching vessels. The NBI endoscopic examination in reflux esophagitis is characterized by microerosions, higher vascularity, and an increased number, dilation, and tortuosity of IPCL at the squamocolumnar junction [48,49,50].

An international prospective randomized controlled trial (RCT) enrolled 123 BE patients randomized to high-definition white-light endoscopy (HD-WLE) with Seattle protocol biopsies or NBI with target biopsies only to compare dysplasia detection. Both NBI and HD-WLE were equally effective in detecting IM (92%). However, NBI performed better for dysplasia detection (30% vs. 21%, *p* = 0.01), and the patients in the NBI group required a lesser number of biopsies (3.6 vs. 7.6 per patient, *p* < 0.0001) [51]. These results would also have financial implications, saving healthcare dollars.

A meta-analysis of 11 studies showed that NBI has a sensitivity and specificity of 0.91 (95% CI: 0.86–0.94) and 0.85 (95% CI: 0.76–0.92) on a per-patient basis for BE diagnosis, respectively. Similarly, NBI was found to have a sensitivity of 0.91 (95% CI: 0.75–0.98) and a specificity of 0.95 (95% Cl: 0.91–0.97) in the diagnosis of high-grade dysplasia (HGD) in BE [52].

ii. **Flexible spectral Imaging Color Enhancement (FICE)**

Flexible spectral imaging color enhancement (FICE) is another virtual chromoendoscopic tool that provides dye-free contrast enhancement of the mucosa and the vessels. NBI utilizes optical filters within the light source, whereas FICE utilizes post-processing techniques such as computed spectral estimation to reconstitute virtual images [53]. The diagnostic finding for GERD in FICE is a triangular indentation that extended from the villiform columnar into the squamous mucosa at the squamocolumnar junction. In a study by Chaiteerakij, FICE had a sensitivity of 77.8%, a specificity of 83.3%, a PPV of 93.3%, an NPV of 55.6%, and an accuracy of 79.2% in detecting minimal mucosal erosions in patients with typical reflux symptoms [45]. However, the inter-observer agreement was low, with a Kappa value of 0.28.

iii. **I-Scan**

**Figure 4:**
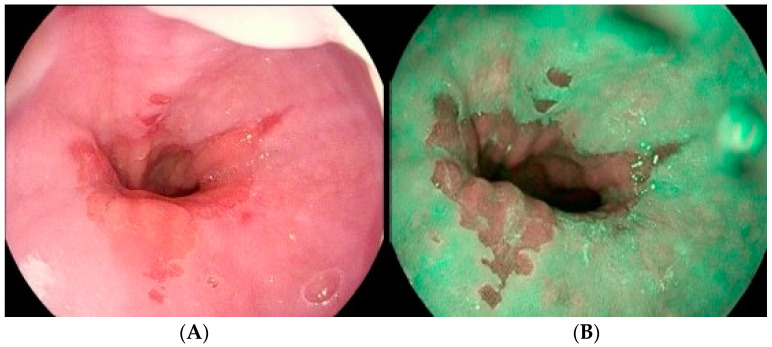
Comparison of white light endoscopy (**A**) and i-scan with an irregular Z line and minimal-change GERD (**B**).

I-scan is a software-based technology that uses optical enhancement techniques to produce high-resolution images to improve the sharpness, hue, and contrast (Figure 4). The images are enhanced from (i) surface enhancement for the mucosa, (ii) contrast enhancement, which adds blue color to the dark areas, and (iii) tone enhancement [54]. Previous studies have reported that the sensitivity, specificity, PPV, and NPV of i-scan in detecting minimal mucosal changes in GERD were 51.35%, 67.33%, 36.54%, and 79.06%, respectively [54,55].

### 3.4. Autofluorescence Imaging

Autofluorescence imaging (AFI) endoscopy utilizes the properties of light-tissue interaction to produce real-time pseudocolor images to facilitate the early diagnosis of neoplasia [56]. It is based on the principle of detecting fluorescence generated from endogenous fluorophores induced by excitation light. This technology helps visualize subtle malignant or dysplastic lesions because of variations in the fluorescence emission between neoplastic and healthy tissue [46,57,58,59,60]. In a multi-center RCT of BE patients, the diagnostic yield of AFI with target biopsies for adenocarcinoma/HGD was 12% compared to 5.3% for conventional endoscopic surveillance with four-quadrant biopsies on a per-patient basis. However, AFI had a low sensitivity of 42% for detecting adenocarcinoma/HGD. Therefore, AFI-targeted biopsies and four-quadrant biopsy protocols are recommended [38].

AFI-III is a newer-generation AFI designed to enhance early neoplasia detection and reduce false-positive rates in BE by targeting fluorescence in malignant cells. In a study by Boerwinkel et al., AFI-III detected high-grade intraepithelial neoplasia (HGIN)/early cancer in 95% of the patients with BE, as opposed to a 47% detection rate by white light endoscopy. However, it had a high false-positive rate of 86% [59].

### 3.5. Confocal Laser Endomicroscopy (CLE)

Confocal Laser Endomicroscopy (CLE) is an optical sectioning technology that allows for submucosal analysis up to 250 μm and provides real-time in vivo cellular imaging to detect BE [57,61]. CLE is available as a probe-based CLE (Cellvizio^®^, Mauna Kea technologies, Paris, France) or as an endoscope-based CLE (Pentax EC 3830FK, Tokyo, Japan) [46]. In a study by Kiesslich et al., patients with chronic GERD (>15 y) or those with known BE for surveillance endoscopy underwent CLE to evaluate for BE and associated neoplasia [62]. The CLE predicted IM with a 90% sensitivity, 94.1% specificity, and 96.8% accuracy. In terms of diagnosing BE-associated neoplasia, it had a 92.9% sensitivity, 98.4% specificity, and 97.4% accuracy. It also had excellent inter- and intra-observer agreement, with mean kappa values of 0.843 and 0.892, respectively [62]. The microvessel density was reported to be significantly higher in the neoplastic Barrett’s when compared with non-neoplastic BE (23.6% vs. 14.2%; *p* < 0.001) [63]. In a meta-analysis of 14 studies with 789 BE patients, CLE had a pooled sensitivity of 89% (95% CI: 0.82–0.94) and a pooled specificity of 83% (95% CI: 0.78–0.86) in the detection of HGD and esophageal neoplasia [39]. Overall, CLE has been proposed as a noninvasive, in vivo method for neoplasm surveillance in BE patients [39].

### 3.6. Esophageal Capsule Endoscopy (ECE)

The PillCam ESO^®^ (Given Imaging Ltd., Yoqneam, Israel) capsule is the esophageal capsule endoscopy (ECE) system that was approved by the Food and Drug Administration (FDA) in 2004 to evaluate esophageal disorders such as GERD and suspected BE. The esophageal PillCam is similar to the small bowel (SB) PillCam regarding the size, shape, and weight of the capsule endoscope (26 × 11 mm, less than 3.0 g). However, there are some technical modifications to make it suitable for esophageal examination. Some of the differences include the short battery life (20 min vs. 12 h in SB pillcam), it capturing a higher number of images per second (18 vs. 2–3 frames per second), and it having a wider angle [64,65].

In a multi-center prospective study of patients with chronic reflux, the diagnostic yield of ECE was comparable to that of EGD. In detecting esophagitis, ECE had a sensitivity of 79%, a specificity of 94%, a PPV of 83%, and an NPV of 92%. For the detection of endoscopically suspected BE, ECE had a sensitivity of 71%, a specificity of 99%, a PPV of 83%, and an NPV of 98%. In summary, ECE had a higher specificity and lower sensitivity for detecting esophagitis and BE [66]. A meta-analysis showed that ECE had a moderate yield in diagnosing BE, with a pooled sensitivity of 77% and a pooled specificity of 86%. ECE had a higher rate of patient preference [40].

### 3.7. Wide-Area Transepithelial Sampling (WATS3D)

Wide-area transepithelial sampling with computer-assisted three-dimensional analysis (WATS^3D^) is an innovative method that reduces sampling errors by employing a brush biopsy device to extensively sample potentially affected mucosa. It aids pathologists in diagnosing dysplasia/neoplasia by generating three-dimensional images of entire crypts using a neural network-based software program. Several large prospective trials, conducted in both academic and community settings, have demonstrated significantly higher detection rates of dysplasia and intestinal metaplasia in the screening and surveillance of Barrett’s esophagus (BE) patients when (WATS^3D^) is used alongside standard Seattle protocol-based forceps biopsies. The WATS^3D^ diagnostic platform has been incorporated into the latest American Society for Gastrointestinal Endoscopy Barrett’s guideline as an adjunct to forceps biopsies, with a conditional recommendation despite a low quality of evidence [67].

In the largest prospective study to date, adding WATS^3D^ to forceps biopsies (FB) increased Barrett’s esophagus (BE) detection by 153% and dysplasia detection by 242% among 12,899 patients in screening or surveillance across 21 community practices [68]. These benefits were observed consistently in both screening and surveillance patients.

Despite the effectiveness of WATS^3D^ in detecting intestinal metaplasia (IM) and dysplasia in BE, several questions remain. Currently recommended as an adjunct to Seattle protocol FBs, randomized trials comparing dysplasia and carcinoma detection rates between FB and WATS^3D^ are crucial, especially in low-risk populations. Further prospective studies are needed to explore the clinical significance and natural history of detected dysplasia. Additionally, the performance of WATS^3D^ in different BE patient groups, such as short-segment versus long-segment BE and those with or without hiatus hernia, warrants investigation.

## 4. Barrett’s Esophagus

Barrett’s esophagus (BE) is a condition resulting from chronic GERD in which the metaplastic columnar epithelium replaces the stratified squamous epithelium from the distal esophagus [69]. BE is recognized as the sole premalignant precursor to esophageal adenocarcinoma (EAC), a highly lethal cancer with a poor prognosis [70]. Esophageal adenocarcinoma (EAC) has shown rapid growth in the United States, increasing six-fold (from 4 to 23 cases per 1 million) between 1975 and 2001 [71]. At the time of diagnosis, over 40% of patients with esophageal adenocarcinoma (EAC) already have distant metastasis [72].

Major gastroenterology societies in the United States and Europe recognize that there is insufficient evidence to support mass screening for BE, as there are no randomized, controlled trials (RCTs) investigating the effectiveness of screening [73,74]. These societies recommend considering BE screening for individuals with chronic (≥5 years) GERD who also have multiple additional risk factors. Typically, the high-risk group includes Caucasian men with longstanding or frequent GERD, along with one or more other risk factors such as an age over 50 years, a smoking history, a waist-to-hip ratio exceeding 0.9, and a family history of BE or esophageal adenocarcinoma (EAC).

In the US, the diagnosis of BE requires the presence of columnar epithelium lining ≥1 cm in the distal esophagus and intestinal metaplasia characterized by goblet cells on biopsy [69,75,76]. Patients with nondysplastic BE have a very low risk of developing esophageal adenocarcinoma (0.1 to 0.4% per year), whereas the risk is higher in patients with high-grade dysplasia (4–8% per year) [77]. Patients with BE undergo surveillance at intervals depending on the length of BE and the presence of dysplasia. Surveillance aims to improve the early detection of dysplasia or esophageal adenocarcinoma to provide timely treatment. Biopsies of the suspected BE segment are required to establish the diagnosis of BE [77]. Seattle biopsy protocol is utilized to obtain random biopsies, which obtains at least four biopsies for every 2 cm segment of suspected BE [69,73,74,78].

The current strategies are deemed inadequate, as the majority of patients diagnosed with esophageal adenocarcinoma (EAC) do not have a prior BE diagnosis, and over 90% have not undergone previous EGD [79,80]. Conventional EGD coupled with histopathological confirmation is considered the definitive method for identifying Barrett’s esophagus (BE). Direct costs include medication administration, monitoring, personnel, and recovery expenses, while indirect costs encompass the need for a companion to escort the patient home and potential work absences due to sedation [81]. Innovations in the space of minimally invasive and noninvasive screening techniques, such as transnasal endoscopy (TNE), esophageal capsule endoscopy (ECE), Cytosponge (Medtronic), EsophaCap (CapNostics), and EsoCheck (Lucid Diagnostics), have shown promising results in clinical trials [70].

## 5. Management

Pharmacological therapy: The cornerstone of pharmacological treatment for GERD involves medications for neutralizing or decreasing gastric acid production. These include antacids, histamine H2-receptor antagonists (H2RAs), and proton pump inhibitors (PPIs). Antacids are primarily used for relieving symptoms as needed, without clear evidence favoring one type over another. Research on an alginic acid formulation from the UK suggests it may offer effective symptom relief compared to other products, although the consistency of the alginate content varies in preparations available in other countries [82]. Proton pump inhibitors demonstrated a notably quicker healing rate (12% per week) compared to H2-receptor antagonists (6% per week), as well as more rapid and comprehensive relief from heartburn (11.5% per week versus 6.4% per week with H2-receptor antagonists) [82].

An 8-week treatment course is necessary for adequate healing, and patients should not be considered non-responders until this period, except in the presence of alarm symptoms. If alarm symptoms are present, upper endoscopy should be conducted within 2 weeks. For patients without alarm symptoms but persistent reflux despite treatment, endoscopy should be performed after 8 weeks, including biopsies of the esophagus to evaluate for eosinophilic esophagitis [83].

Reflux inhibitors, prokinetics, acupuncture, and hypnotherapy are under investigation for GERD treatment. Prokinetics, such as metoclopramide and domperidone, have shown effectiveness in specific GERD cases but are limited due to associated central nervous system side effects and QT prolongation risks.

Vonoprazan works by competing with potassium for its binding site, which prevents potassium from binding and, as a result, inhibits the secretion of gastric acid. The potassium-competitive acid blocker vonoprazan was non-inferior and superior to the PPI lansoprazole in the healing and maintenance of the healing of erosive esophagitis. This benefit was seen predominantly in more severe erosive esophagitis. More studies are needed in the future to evaluate its role in GERD [84].

Lifestyle: Typical recommendations for GERD include weight loss for overweight individuals, raising the head of the bed, quitting tobacco and alcohol, avoiding late-night meals and snacks before bedtime, remaining upright during and after meals, and eliminating foods that may exacerbate reflux symptoms such as coffee, chocolate, carbonated beverages, spicy foods, acidic foods like citrus and tomatoes, and high-fat foods [85]. The evidence supporting these recommendations is limited and variable, often based on small, uncontrolled studies and rarely as the sole intervention, which complicates definitive recommendations. However, several randomized controlled trials have demonstrated an improvement in nocturnal GERD symptoms and nocturnal esophageal acid exposure with head-of-bed elevation or sleeping on a wedge. The timing of food intake can also affect GERD symptoms. A short interval (<3 h) between eating and bedtime or lying supine is associated with increased GERD symptoms and the need for medication.

Diet: Various foods have different effects on LES pressure. Coffee, caffeine, citrus, and spicy food had little to no effect on LES pressure [86,87]. However, the irritant effects of them could evoke GERD symptoms.

Smoking cessation was shown to improve GERD symptoms in a large cohort study [88]. Additionally, El Serag et al. showed that patients who successfully quit smoking for a year had a 44% improvement in GERD symptoms, compared to an 18% improvement in those who continued to smoke [89].

## 6. Endoscopic Interventions for GERD

Procedural treatments for GERD include surgical or endoscopic interventions. The primary goal of these interventions is to restore the physiologic equivalent to the natural LES [90,91]. However, these interventions face challenges in achieving the normal functionality of the gastroesophageal junction (GEJ) along with the iatrogenic harm associated with these procedures [91]. Nissen fundoplication has been shown to be very effective and has excellent long-term outcomes. It mainly involves wrapping up the intra-abdominal esophagus (360° complete wrap) using the mobilized gastric fundus to strengthen the LES [90]. The modifications of Nissen fundoplication include Dor fundoplication with a 180° wrap and Toupet fundoplication with a 270° wrap. Endoscopic interventions have emerged as a minimally invasive treatment option for patients not responding to medical treatment and who do not want surgery. The various endoscopic therapies that have been developed include electrical stimulation, radiofrequency therapy, transoral incisionless fundoplication, endoluminal plication, the injection of biopolymers, endoscopic mucosal resection (EMR), and endoscopic opposition devices (Table 3 and Table 4).

### 6.1. Electrical Stimulation of the LES

EndoStim^®^ (EndoStim BV, The Hague, The Netherlands) is based on the principle of LES electrical stimulation (EST), which has been shown to increase LES pressure significantly and improve symptoms such as heartburn and regurgitation [2,105]. Most of the studies involved the placement of this device laparoscopically, which showed a sustained improvement in GERD health-related quality of life (HRQL), a decreased use of daily GERD medications, and a reduction in distal esophageal acid exposure [106]. In a small study (n = 6) by Banerjee et al., endoscopic placement of the device was performed with a 3 cm submucosal tunnel, and one patient had a premature lead dislodgement. All of the other five patients had successful EST with a significant increase in LES pressure. There were no EST-related adverse events and no effect on the swallow-induced LES relaxation or cardiac rhythm [92]. EST has the potential to be utilized more widely in the coming years. More studies are needed on the endoscopic implantation technique.

### 6.2. Anti-Reflux Mucosectomy

Anti-reflux mucosectomy (ARMS), first reported in 2014 by Inoue et al., is a new endoscopic treatment for patients with refractory GERD. The procedure aims to achieve fundoplication by performing a hemi-circumferential mucosal resection of cardia around the GEJ. The healing process creates scar formation, resulting in the gastric cardia’s contraction and narrowing the GEJ opening, thereby reducing acid reflux [2,22,23,107]. This concept of ARMS originated from BE patients who underwent circumferential mucosal resection for short-segment HGD, which significantly improved their GERD symptoms [2]. In a pilot study, ARMS was performed on ten patients with refractory GERD and led to a significant improvement in GERD symptoms [108]. The study reported a significant improvement in the GERD symptoms, with an improvement of the mean regurgitation score (a component of the DeMeester score) from 2.5 to 0.3 (*p* = 0.002) and a similar improvement in the heartburn score from 2.7 to 0.3 (*p* = 0.001) [108]. In a retrospective study by Sumi et al., for patients with PPI-refractory GERD who underwent ARMS, there was a significant reduction in the acid exposure time (from 20.8 to 6.9 min, *p* < 0.01) and an improvement in the DeMeester score (from 64.4 to 24.9, *p* < 0.01), and up to 50% of the patients were able to discontinue PPIs at the end of two months [93]. However, the reduction in proximal reflux episodes did not reach statistical significance (*p* = 0.08). Furthermore, transient esophageal stenosis was reported after 2–3 weeks in 13 patients (11.9%) requiring balloon dilation [93]. In a meta-analysis that included ten studies with 307 patients who underwent ARMS for refractory GERD, the technical success rate was 97.7% (95% CI, 94.6–99.0), with a clinical response rate of 80.1% (95% CI, 61.6–91.0) [109]. After ARMS, there was a significant improvement in the GERD-HRQL (mean difference MD = 14.9, *p* < 0.001) and GERD questionnaire (GERD-Q) scores (MD = 4.85, *p* < 0.001), with a significant reduction in the mean acid exposure time (MD = 2.39, *p* = 0.01). The overall rate of adverse events (AE) was 17.2% (95% CI, 13.1–22.2), with dysphagia (11.4%) and bleeding (5%) being the most common complications. More RCTs are required to illustrate long-term benefits prior to broader recommendations.

### 6.3. Radiofrequency Therapy

Radiofrequency therapy using the Stretta system^®^ (Mederi Therapeutics, Norwalk, CT, USA) is one of the most widely studied endoscopic anti-reflux procedures. This procedure was FDA-approved in 2000 [110]. It is indicated in patients with breakthrough GERD symptoms despite escalating doses of PPI, patients who cannot tolerate PPI, patients who do not wish to undergo anti-reflux surgery, or those who are poor surgical candidates [111]. Patients with hiatal hernia >3 cm, those with an LES pressure <5 mmHg, those who did not respond to PPI therapy, or those with negative pH impedance studies are not good candidates for radiofrequency therapy [112]. This procedure delivers controlled radiofrequency energy with four needle electrodes to the LES, GEJ, and gastric cardia using a four-channel radiofrequency generator [113,114,115]. This regenerates the muscularis propria and thereby increases the thickness of the muscular layer. This is considered to improve the function of LES as a barrier to decrease the number of inappropriate LES relaxations and thereby reduce acid reflux. Stretta^®^ operates at low power settings (465 mHz, 2–5 W per channel, 80 V maximum), which results in a lower temperature (65 °C–85 °C) in the muscularis propria with the goal of muscle regeneration, as opposed to radiofrequency ablation, which uses higher temperatures to ablate the diseased mucosa [2,111].

In an RCT by Corely et al., comparing Stretta^®^ to sham procedures, there was no difference in the reduction in PPI use; however, the study found a significant improvement in GERD-HQRL scores in the Stretta^®^ group (61% vs. 33%, *p* = 0.03) [116]. Another RCT by Art et al. showed a significant improvement in the symptom scores and a decrease in GEJ compliance three months after the Stretta^®^ procedure. However, there were no changes in the acid exposure or LES pressure. The authors also noted that the GEJ compliance reversed back to pre-Stretta levels with the administration of Sildenafil, suggesting that the decreased compliance could be secondary to altered neuromotility rather than fibrosis [117]. The meta-analysis by Fass et al. showed that patients who underwent Stretta^®^ had a significant improvement in mean HRQL scores (−14.6, 95% CI −16.48, −12.73), an improvement in pooled heartburn scores (−1.53, 95% CI −1.97, −1.09), a reduction in the use of daily PPI (by 51%), a reduction in erosive esophagitis (by 24%), and a reduction in mean esophageal acid exposure (−3.01, 95% CI −3.72, −2.30) [94,118]. The overall adverse events from Stretta^®^ were less than 1%. Long-term studies have shown positive results on the durability of the Stretta^®^, with up to 25% of the patients being able to be off their PPI for 2–8 years [119,120,121]. Few studies have shown that Stretta^®^ may benefit patients with GERD symptoms after bariatric and anti-reflux surgeries [122,123].

In general, Stretta^®^ is considered a safe procedure in mild-to-moderate GERD patients who have failed or had a partial response to medical management. Stretta^®^ should be considered an alternative therapeutic option in managing GERD, especially in patients seeking non-surgical options [2].

### 6.4. Transoral Incisionless Fundoplication

Transoral Incisionless Fundoplication (TIF) is an endoscopic procedure in which the LES is reconstructed to restore the angle of His to improve the LES barrier function [111]. The devices that are used to perform TIF include the EsophyX^®^ device (EndoGastric Solutions, Redmond, WA, USA) or the Medigus Ultrasonic Surgical Endostapler system (MUSE™, Medigus Ltd., Omer, Israel). The new gastroesophageal flap valve is created within the stomach by performing full-thickness serosa-to-serosa plications, including the muscle layer [124]. TIF is a promising and safe option in patients with refractory GERD with typical symptoms. The contraindications of TIF include patients with atypical symptoms, extraesophageal manifestations, LA grade C or D erosive esophagitis, BE, and esophageal pathology or surgery [125].

**Figure 5:**
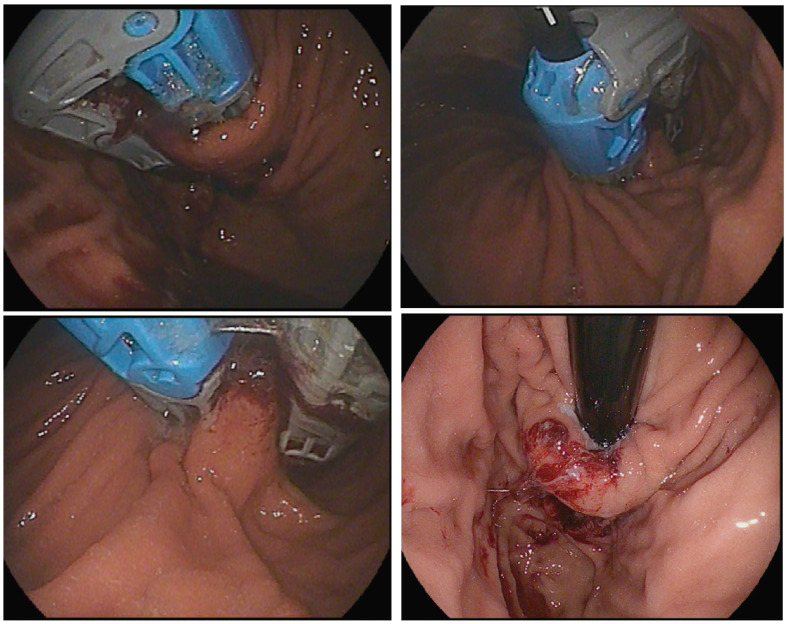
Transoral rotational esophagogastric fundoplication with gastrogastric plications placed proximal to the Z-line.

Esophyx^®^: This was first introduced in 2005 and has undergone two modifications. The first modification in 2007 resulted in TIF 1.0 and was replaced with TIF 2.0 in 2009, with better results [126]. The EsophyX^®^ device constructs a 3–5 cm long omega-shaped valve by deploying polypropylene fasteners in a 270° circumferential pattern around the GEJ (Figure 5).

Several randomized trials have been performed to demonstrate the efficacy of TIF over PPIs or sham interventions [124,126,127,128]. The RESPECT study (Randomized EsophyX2 versus Sham, Placebo-Controlled TIF) included patients with persistent regurgitation despite the daily use of PPI. The patients were randomized to TIF combined with placebo medication (n = 87) or to a control arm with a sham operation and PPI therapy (n = 42) for six months [129]. Overall, the patients in the TIF group had better outcomes than the control group. Troublesome regurgitation was eliminated in a higher proportion of patients in the TIF group compared to the control group (67% vs. 45%, *p* = 0.023). There was also a significant improvement in the esophageal pH from 9.3% to 6.3% (*p* < 0.001) after the TIF procedure, whereas there was no improvement in esophageal pH in patients in the control group [129]. The TEMPO trial showed that the TIF was more effective in treating GERD when compared with those treated with a maximum standard dose of PPI at six months [130]. Patients in the TIF group had higher rates of improvement in regurgitation and extraesophageal symptoms (62% vs. 5%, *p* = 0.009), and 90% of patients were off PPI. In a meta-analysis of 18 studies in 963 patients, the pooled response rate to TIF compared to that to the PPI or sham was 2.44 (95% CI 1.25–4.79, *p* = 0.0009). However, there was no significant improvement in the esophageal acid exposure time or acid reflux episodes, and most patients resumed PPI at lower doses during the long-term follow-up. The rate of severe adverse events, such as gastrointestinal bleeding or perforation, was 2.4% [131].

In a study of daily PPI-dependent GERD patients who underwent TIF and followed up for six years, about 50% stopped PPI, and 86% stopped using high-dosage PPI. The results obtained at 36 months were maintained at a 6-year follow-up. Failure or unsuccessful outcomes mainly occurred between 6 and 12 months [132].

MUSE™: The Medigus Ultrasonic Surgical Endostapler (MUSE™) (Medigus, Omer, Israel) is another stapling device used to perform transoral partial fundoplication [22]. FDA has cleared the MUSE™ System for marketing. The device contains a miniature video camera, an ultrasound transducer, and an endo-stapler. Similar to the surgical Dor-Thal fundoplication surgery, the MUSE™ system creates a 180-degree anterior fundoplication by stapling the fundus of the stomach to the esophagus under ultrasound guidance [2,22,107]. In a multi-center prospective clinical study of 66 patients, 73% reported improved GERD-HRQL scores, and 64.6% stopped taking PPIs at six months. Adverse events such as upper GI bleeding, esophageal leak, pleural effusion, pneumomediastinum, and pneumoperitoneum occurred at a higher frequency during the initial use of the device (8 adverse events in the first 24 subjects). Protocol and device changes were implemented after an interim review of these early adverse events, which reduced the adverse events, and no additional cases of pneumomediastinum or leak were reported after that [96]. In a long-term follow-up study, 83.8% of patients who underwent the MUSE™ procedure were off PPI at six months, and 69.4% remained off PPI at four years. The mean GERD-HRQL score improved by 24 points from 29.1 ± 5.6 to 5.3 ± 5.8 (*p* < 0.01), along with a significant reduction in the dose of daily PPI by approximately 55 omeprazole equivalents from 66.1 (±33.2) to 12.8 (±19.4) at four years [96]. Although MUSE™ is effective, further randomized trials with long-term outcomes are needed.

Current evidence substantiates the TIF procedure as a safe, viable, effective, and promising therapeutic substitute for medical and surgical therapy. Despite encouraging short- and medium-term outcomes, future studies are needed to follow up on the long-term efficacy, especially for the MUSETM technique.

### 6.5. Endoscopic Full-Thickness Plication (GERDx™)

The GERDx™ (G-SURG GmbH, Seeon-Seebruck, Germany) is another minimally invasive endoscopic option for patients with GERD introduced in 2014. Compared with other Endoscopic full-thickness plication devices, GERD-X is simpler and less cumbersome to operate, with a shorter procedure time. The device includes an applicator, tissue retractor, and suture system. First, the tissue retractor is advanced into the gastric wall within 1 cm of the GEJ. After the gastric wall is retracted into the instrument, the suture implant is deployed to create full-thickness plication [2,22,133]. In a prospective single-arm trial of 40 GERD patients who underwent the GERDx™ procedure with a 3-month follow-up. there was a significant improvement in the mean DeMeester score (from 46.48 ± 30.83 to 20.03 ± 23.62) and the mean GI QOL index (from 92.45 ± 18.47 to 112.03 ± 13.11). Four patients had serious adverse events, which included GEJ hematoma, Mallory Weiss tear, pneumonia, and intractable post-procedure pain requiring laparoscopic suture removal [134]. In an RCT by Kalapala, 70 patients with PPI-dependent GERD were randomized to either the GERD-X arm or sham procedure and followed up for 3 months [135]. The patients in the GERD-X group had a higher proportion of patients with >50% improvement in the GERD-HRQL when compared with the sham group (65.7% vs. 2.9%, *p* < 0.001). Also, 62% of the patients who underwent GERD-X could come off PPI at 12 months, compared with 11% in the sham group. There were no serious adverse events from the procedure.

### 6.6. Emerging Gastroesophageal Junction (GEJ)-Altering Techniques

Endoscopic band ligation (EBL) has been used to treat large esophageal varices since 1986. EBL was also thought to strengthen the GEJ, based on the serendipitous findings of the improved appearance of GEJ and the improvement in reflux symptoms. A pilot study was first conducted in 2013 on patients with refractory GERD, which showed better quality-of-life scores in the EBL group. In an RCT by Saleem et al. in 2017, a total of 150 patients with refractory GERD were randomized either to the EBL group or the control group. Patients in the EBL group received four quadrants banding at the GEJ in one session (N = 75), and the patients in the control group were treated with optimized doses of PPI (N = 75) [103]. At one year of the follow-up, patients in the EBL group had a significant improvement in the GERD-QoL score (by 43%) and a significant reduction in reflux episodes (11 vs. 218, *p* < 0.001) and the symptom index (0.22 vs. 0.7, *p* < 0.05) when compared to the control group. Furthermore, there were no major adverse events [103].

**Figure 6:**
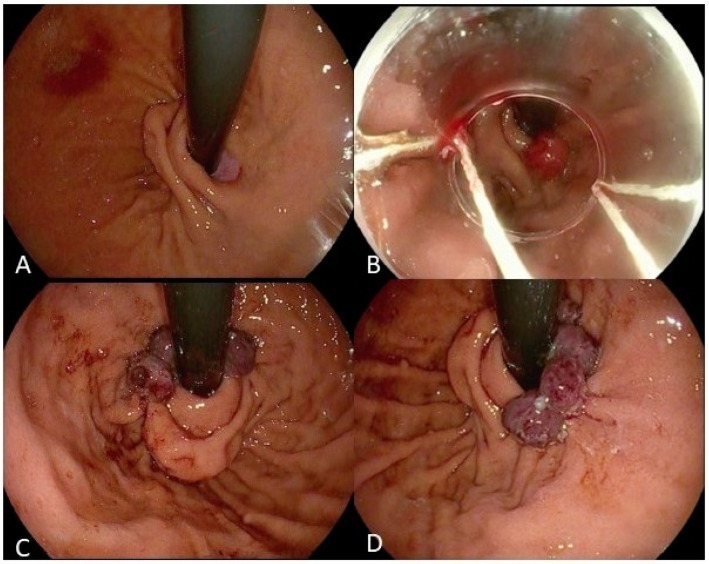
Cardia Ligation Endoscopic Anti-Reflux (CLEAR) procedure: (**A**) The assessment of cardia. (**B**) Capture of mucosa at the level of the gastroesophageal junction with the band ligation device. (**C**,**D**) Capture of mucosa one by one, oriented towards the lesser curvature of the stomach.

Another modification of this method is to place bands at proximal gastric cardia just below the GEJ (Figure 6), which heals by the mucosal contraction and narrowing of GEJ, similar to ARMS. In an attempt to provide a less invasive treatment, Hu et al. developed peroral endoscopic cardial constriction (PECC), in which the cardia diameter is narrowed, thereby preventing the reflux of stomach contents. This study assessed 13 GERD patients who underwent PECC and found that the GERD-HRQL score improved from 19.9 ± 7.9 to 4.5 ± 4.3 and 5.7 ± 5.1 at 3 and 6 months after the PECC procedure, respectively. In addition, there was a significant decrease in the esophageal acid exposure time with a pH below 4, significantly decreasing from 35.6 ± 26.2 before the PECC treatment to 7.9 ± 13 and 4.7 ± 3.8 at 3 and 6 months after PECC. No severe treatment-related complications were reported during this study [97]. This procedure has also been termed as the cardia ligation endoscopic anti-reflux (CLEAR) procedure. The long-term efficacy of the CLEAR procedure is unknown, and some patients might need to undergo repetitive procedures depending on the recurrence of symptoms.

The resection and plication (RAP) anti-reflux procedure is another innovative endoscopic procedure involving semi-circumferential mucosectomy and full-thickness plication of the LES and cardia. In a study by Benias et al., 10 patients with refractory GERD underwent RAP procedure, with a median follow-up of 9 months (range: 5 to 24 months). The study patients significantly improved their GERD-HRQL scores, and 80% of the patients stopped using their daily PPI [104].

### 6.7. Suturing, Plicating Devices

i. **Endoscopic Suturing Device:**

The Endoscopic Suturing Device comprises a flexible Sew-Right apparatus, a pliable Ti-Knot tool, and an external accessory channel. The Sew-Right and Ti-Knot devices are passed through the external accessory channel. A single suture loop is created using the Sew-Right device, which plicates the two adjacent areas in the proximal stomach [4]. However, initial studies reported an early loss of sutures, with only 5% retaining sutures at six months. In addition, no significant improvement was found in quality-of-life scores or reflux esophagitis or esophageal acid exposure based on the median pH < 4 per 24 h (9.9% vs. 12.3%, *p* = 0.60) when compared with the baseline [98]. Also, Schilling et al. showed a similarly poor outcome of early suture loss with no clinical improvement with this method [136].

ii. **BARD EndoCinch™ (Murray Hill, New Jersey, USA):**

Swain and Mills introduced the BARD endoluminal gastroplication procedure in 1986, subsequently receiving FDA approval in the year 2000. It is a popular and well-studied procedure often compared with surgical fundoplication [137]. The EndoCinch procedure uses a sewing capsule attached to the distal tip of an endoscope to create partial-thickness pleats through a series of sutures at the gastric cardia [98]. Ozawa et al. conducted a multi-center prospective, open-labeled trial in 48 GERD patients undergoing endoluminal gastroplication using the EndoCinch™ system. During a 24-month follow-up period, the rate of the complete resolution of heartburn symptoms ranged from 54 to 66%, and the rate of the successful discontinuation of PPI or H2 receptor antagonists ranged from 65% to 76%. No serious adverse events were observed [138]. However, a long-term follow-up study has shown the loosening of sutures from a lack of full-thickness fundoplication and poor mucosa apposition with sutures even after enhanced modification [139,140]. EndoCinch ™ fails to show long-term benefits for most patients with GERD, and it is shown to be inferior to surgical fundoplication [141].

iii. **NDO Plicator:**

Chuttani et al. conducted a pilot study in 2003 using a full-thickness suturing transmural plicator to address the weakness of EndoCinch [142]. Thirty chronic GERD patients across seven sites were followed for approximately five years. This long-term follow-up study reported patients who were PPI-dependent prior to treatment. A total of 67% remained off daily PPI therapy at 60 months. During a 5-year follow-up, the median GERD HRQL scores showed a significant improvement from the baseline scores (10 versus 19, *p* < 0.001) [143]. Jeansonne et al. compared the effectiveness of endoscopic full-thickness plication (FTP) and endoscopic radiofrequency treatments for patients with GERD. In the FTP group, patients with moderate-to-severe heartburn decreased from 53% to 43% (*p* = 0.3), and PPI use decreased from 95% to 43% (*p* = 0.01). The percentage of time during which the pH remained < 4 decreased from 10.0% to 6.1% (*p* = 0.05). Also, a dramatic reduction in regurgitation was noted in the FTP group [144]. In a prospective RCT by Rothstein et al., the patients were randomly assigned to the endoscopic FTP (n = 78) and sham group (n = 81). The intention-to-treat analysis showed patients achieving ≥50% improvement in GERD-HRQL scores, significantly higher in the FTP group (56%) than in the sham group (18.5%) at three months (*p* < 0.001). Similarly, it showed that the patients in the FTP groups had higher rates of PPI cessation when compared with the sham group (50% vs. 24%, *p* = 0.002). No perforation or deaths were reported [100]. The device has not been available since June 2008 due to poor financial performance [139].

### 6.8. Anti-Reflux Device (ARD)

The Syntheon Anti-reflux Device (Syntheon, Miami, FL, USA) used a titanium implant inserted into the cardia to create a full-thickness serosa-to-serosa apposition. The ARD™ instrument can be passed over the gastroscope and operated independently. This is in contrast to the NDO plicator passed through the endoscope. The gastric wall is pulled into the ARD using a tissue retractor which is passed through the biopsy channel, and a full-thickness pleat is created by deploying the titanium implant. In a multi-center clinical trial, patients with severe GERD (N = 57) were treated with ARD; at six months of follow-up, 63% of the patients stopped all PPI or other antisecretory medications, and 79% of the study subjects reported an improvement in GERD-HRQL scores of 50% or more [101]. On follow-up endoscopy, the implants were all found in place, and one gastric perforation occurred, requiring surgical repair. ARD has not been commercially available [4].

### 6.9. The His-Wiz Anti-Reflux Procedure

This novel overtube-based endoscopic device, His-Wiz (Apollo Group/Olympus Optical, Tokyo, Japan), allows for simple and rapid full-thickness infrasphincteric plication to improve the gastroesophageal barrier [102]. A clinical trial on seven patients showed subjective heartburn scores and an objective improvement in pH testing on one-year follow-up [102]. However, the results tend to decline over time. The effectiveness and permanence of this procedure are still not fully understood; hence, this device is not commercially available.

### 6.10. Injectable Agents

Injectable agents were introduced as an attractive and simple option for the management of GERD, where a bulking material is injected or implanted endoscopically at the GEJ to improve the barrier to prevent reflux. However, they were largely unsuccessful due to poor efficacy and safety records.

Enteryx™ (Microvasive Endoscopy, Boston Scientific Corp, Natick, Mass) is a non-resorbable, non-viscous liquid copolymer that is injected into the LES. It solidifies rapidly in situ and modifies the compliance of the cardioesophageal junction, thereby preventing the reflux of gastric contents. Clinical trials showed a 50% PPI reduction in more than 80% of the study population with a significant improvement in GERD [145]. However, no improvement was seen in LES pressure, esophagitis, and acid exposure in the esophagus [146]. The manufacturer recalled Enteryx™ in 2005 due to serious adverse events, including deaths related to inadvertent transmural injections of the product into structures surrounding the esophagus.

The Gatekeeper™ Reflux Repair System (Medtronic Inc., Minneapolis, MN, USA) uses a polyacrylonitrile-based hydrogel prosthesis endoscopically introduced into the submucosa for LES augmentation to prevent reflux. In a study by Fockens et al., PPI-responsive GERD patients underwent the procedure and significantly improved GERD HRQL scores and LES pressure at six months in patients who were not on PPI therapy [147]. However, in a follow-up RCT comparing the Gatekeeper with a sham procedure, an interim analysis at six months showed no difference between the two groups. However, there were serious but infrequent adverse events such as severe chest pain, esophageal perforation, and pulmonary infiltrates [148]. Subsequently, the manufacturer voluntarily withdrew the product due to a lack of efficacy and reported complications [148,149].

Durasphere^®^ (Carbon Medical Technologies, MN USA) is an FDA-approved injectable bulking agent for treating intrinsic bladder sphincter deficiency-related stress incontinence. In a pilot study by Ganz et al., 90% of the patients with mild–moderate GERD had a 50% reduction in PPI use at a 12-month follow-up, along with an improvement in the DeMeester score. No significant adverse events were reported in the study [150]. Currently, Durasphere^®^ is not FDA-approved for the management of GERD [151].

The final injectable agent is Plexiglas-polymethylmethacrylate (PMMA) beads, used in cosmetic treatments as a biologically inert filler [149]. The procedure involves the endoscopic implantation of PMMA into the LES submucosa.

In the only study by Feretis et al., there was a significant decrease in the symptom severity score and esophageal acid exposure time (*p* < 0.05), with no procedure-related severe complications [149,152]. It has not been FDA-approved for GERD management [149].

Though the endoscopic injectables theory on its mechanism of action appears reasonable, it comes with questions regarding its safety. Studies have demonstrated poor outcomes and serious adverse events related to accidental injections into adjacent structures. Also, it is not certain that passive resistance on the lower esophageal sphincter could truly affect GERD. More research is needed before these technologies are used as a treatment option.

## 7. Future Directions

Significant advancements have been made in endoscopic technology for the management of GERD over the last two decades. The endoscopic therapies currently available in the US include TIF, radiofrequency therapy, and anti-reflux mucosal resection. Gastric plication devices such as GERDx are available in Europe. The endoscopic therapies were not studied in patients with a hiatal hernia >2 cm because of the risk of failure. Combined TIF and hiatal hernia repair (cTIF) is being evaluated for patients with large hiatal hernia (>2 cm). This is a combined endoscopic and surgical approach, where the surgeon reduces the hiatal hernia, and the gastroenterologist performs the TIF. An ongoing randomized trial is comparing cTIF and laparoscopic Nissen Fundoplication (NCT04795934). The current short-term data for TIF and Stretta show they effectively decrease GERD symptoms and PPI use. However, the long-term data are lacking, which is one of the challenges for these procedures being commonly adopted in routine clinical practice. Also, there needs to be a development of endoscopic treatment options for treating GERD in special populations such as those with sleeve gastrectomy and patients with anatomic issues.

## 8. Conclusions

Newer advanced endoscopic imaging and intervention techniques can improve the diagnostic accuracy of GERD and its complications. Also, it improves the yield of targeted biopsy samples by detecting areas of abnormal mucosal or vascular patterns, which would decrease the total number of biopsies and unnecessary biopsies from non-dysplastic areas. However, these imaging devices are available only in select academic and research institutions due to the need for more training and additional equipment. A significant proportion of patients do not completely respond to the PPI and do not want to undergo surgical intervention. This potential patient group might be candidates for the newer endoscopic interventions that offer a minimally invasive option. The choice of procedure depends on the patient’s characteristics and preferences and the availability of local expertise. We still need more randomized trials to compare pharmacological, endoscopic, and surgical interventions to offer personalized care for GERD.

## Figures and Tables

**Table 1:** Major GERD phenotypes.

GERD Syndrome	Features
Non-erosive or endoscopy-negative reflux disease (NERD)	When assessed through physiological testing, it closely resembles mild esophagitis. It shares characteristics with GERD hypersensitivity and functional heartburn when evaluated based on symptoms.
GERD hypersensitivity	Conceptually distinguished by whether symptoms are positively or negatively associated with reflux on testing; these entities can be clinically identical.
Functional heartburn	
Erosive esophagitis, low grade (LA grade A or B)	LA grade A esophagitis can be found in about 6% of asymptomatic controls, making it a non-specific finding.
Erosive esophagitis, high grade (LA grade C or D)	Grossly abnormal EGJ function with supine reflux and abnormal esophageal acid clearance. Usually associated with hiatus hernia.
Barrett’s esophagus	Endoscopic spectrum from intestinal metaplasia at the EGJ to short-segment to long-segment (>3 cm) Important biological spectrum from non-dysplastic metaplasia to low-grade dysplasia and high-grade dysplasia
Reflux chest pain syndrome	“Non-cardiac” chest pain along with physiological evidence of GERD or typical reflux symptoms is much more amenable to GERD therapy than chest pain without these features.
Regurgitation-dominant reflux disease	Indicative of a grossly incompetent EGJ barrier with large-volume reflux and the need to differentiate from rumination and achalasia.
Laryngopharyngeal reflux (LPR) Chronic cough	While reflux may contribute, it is seldom the primary pathophysiology; typically, significant cofactors are involved. Strongly influenced by neuronal hypersensitivity and responds better to GERD therapy when accompanied by typical reflux symptoms.

**Table 2:** Endoscopic imaging for GERD diagnosis.

Endoscopic Imaging Test	Study	Results
Magnification Endoscopy	Retrospective, N = 500 [35]	Improvement in the detection of pathology, OR 1.87 (95% Cl 1.11–3.12)
Chromoendoscopy	Meta-analysis, N = 843 [36]	Increased diagnostic yield for dysplasia in BE by 34% (95% Cl 20−56%)
Narrow-Band Imaging (NBI)	Meta-analysis, N = 502 [37]	For specialized intestinal metaplasia: Sensitivity = 0.91 (95% CI 0.86–0.94) and Specificity = 0.85 (95% Cl 0.76–0.92) For high-grade dysplasia: Sensitivity = 0.91 (95% CI 0.75–0.98) Specificity = 0.64 (95% Cl 0.59–0.68)
Autofluorescence Imaging (AFE)	Multi-center randomized controlled trial, N = 130 [38]	The diagnostic yield of AFE was better when compared with conventional endoscopy (12% vs. 5.3%).
Confocal Laser Endomicroscopy (CLE)	Meta-analysis, N = 789 [39]	For the detection of neoplasia in BE, a pooled sensitivity of 89% (95% Cl 0.82–0.94) and a pooled specificity of 83% (95% Cl 0.87–0.90)
Wireless Esophageal Capsule Endoscopy	Meta-analysis. N = 618 [40]	For the diagnosis of BE, the pooled sensitivity and specificity were 77 and 86%, respectively.

BE—Barrett’s esophagus; OR—Odds ratio; CI—Confidence Interval.

**Table 3:** Advanced Endoscopic treatments for gastroesophageal reflux disease (GERD).

Method	Treatment Name
Radiofrequency ablation	Stretta system^®^
Endoscopic fundoplication	Transoral incisionless fundoplication (EndophyX)
	Endoscopic full-thickness plication (GERDx)
	Medigus ultrasonic surgical endostapler (MUSE)
Endoscopic mucosal resection	Anti-reflux mucosectomy (ARMS)
	Endoscopic submucosal resection for GERD (ESD-G)
GEJ-altering Techniques	Endoscopic band ligation
	Peroral endoscopic cardial constriction (PECC)
	Resection and plication (RAP) (OverStitch)
Suturing, plicating devices	NDO plicator
	The BARD EndoCinch ™ (C.R. Bard Inc., Murray Hill, NJ, USA)
	The Endoscopic Suturing Device
Electrical stimulation of LES	EndoStim^®^ (EndoStim BV, The Hague, The Netherlands)
Newer techniques (not in market)	The His-Wiz Anti-reflux Procedure
	Anti-reflux Device (Syntheon, Miami, FL, USA)

**Table 4:** Important Studies in Endoscopic Treatment for GERD.

Procedure Name	Evidence	Quality-of-Life Index Improvement	Discontinuation of PPI Use	Improvement in DeMeester Score
Electrical stimulation of LES	Single-center, feasibility study with 6 patients [92]	Not Reported	Not Reported	Not Reported
Anti-reflux mucosectomy	Retrospective study with 109 patients [93]	Not Reported	40–50% patients discontinued PPI at 6–12 months	Improved by 24.9 (SD 36.0) in 2 months
Radiofrequency Ablation (Stretta)	Meta-analysis of 28 studies with 2468 patients [94]	Mean GERD-HRQL improved by −14.6 (95% Cl −16.48 to −12.73)	51% patients discontinued PP	Improved by −13.79 (−20.01, −7.58)
Transoral incisionless fundoplication	Meta-analysis of 32 studies with 1475 patients [95]	Mean GERD-HRQL -improved by 17.72 (95% Cl 17.31–18.14)	89% patients discontinued PP	Improved with mean difference of 10.22 (95% Cl 8.38–12.12)
Medigus Ultrasonic Surgical Endostapler	Multi-center prospective trial with 66 patients [96]	Mean GERD-HRQL-Improved by −9.0 (SD 9.1) at 6 months	64.6% patients discontinued PPI at 6 months	Not Reported
Endoscopic Full-Thickness plication (GERDx™)	Prospective study with 40 patients [97]	Mean GIQLI- improved significantly by 112.03 (SD ± 13.11) at 3 months	63.3% patients discontinued PPI at 3 months	Improved by −20.03 (SD ± 23.62) at 3 months
Wilson–Cook Endoscopic Suturing Device	Prospective study with 20 patients [98]	50% of patients reported an improved GERD-HRQL score but did not achieve statistical significance	10% patients reduced PPI use at 6 months	No difference in median pH < 4/24 h (9.9% vs. 12.3%; *p* = 0.60) at 6 months
BARD EndoCinch™	Double-blind, randomized, sham-controlled trial with 60 patients [99]	Improvement in SF-20 at 6 and 12 months	68% patient reduced PPI use by ≥50% at 12 months	Not Reported
NDO Plicator	Multi-center, randomized, patient-blinded, sham-controlled trial with 159 patients [100]	Significant improvement in mean GERD-HRQL by 12.5 (SD 11.1) at 3 months	57% of patients with complete PPI cessation at 3 months	Improved by median of −28 (18.42) at 3 months
Anti-reflux Device	Multi-center study with 70 patients [101]	Mean GERD-HRQL improved to 69% at 6 months	63% of patients discontinued PPI at 6 months	Not Reported
His-Wiz Anti-reflux Procedure	Prospective pilot study with 7 patients [102]	Not Reported	57.14% patients off PPI	Not Reported
Endoscopic Band ligation	Single-center prospective study with 150 patients [103]	Mean GERD-HRQL score improved by 14.7 (SD 3.9) at one year	Not Reported	Not Reported
Peroral endoscopic cardial constriction	Prospective study with 13 patients [97]	Mean GERD-HRQL score improved by 4.46(SD4.31) and 5.69(SD5.07) at 3 and 6 months	Not Reported	Improvement by 16.97 (SD ± 12.76) and 20.32 (SD ± 15.22) at 3 and 6 months
Resection and plication	Prospective study with 10 patients [104]	GERD-HRQL score showed absolute reduction of 22.3 (95% CI 19.3–25.3) at 9 months	80% stopped using PPI at 9 months	Not Reported

GERD-HRQL—Gastroesophageal disease health-related quality of life, GIQLI—Gastrointestinal Quality of Life index, SF-20—20-item Short-Form Health Survey, SD—Standard Deviation, PPI—proton pump inhibitors.

## References

[1] El-Serag H.B., Sweet S., Winchester C.C., Dent J. (2014). Update on the epidemiology of gastro-oesophageal reflux disease: A systematic review. Gut.

[2] Kushner B.S., Awad M.M., Mikami D.J., Chand B.B., Wai C.J., Murayama K.M. (2020). Endoscopic treatments for GERD. Ann. N. Y. Acad. Sci..

[3] Richter J.E., Rubenstein J.H. (2018). Presentation and Epidemiology of Gastroesophageal Reflux Disease. Gastroenterology.

[4] Vassiliou M.C., von Renteln D., Rothstein R.I. (2010). Recent advances in endoscopic antireflux techniques. Gastrointest. Endosc. Clin. N. Am..

[5] Eusebi L.H., Ratnakumaran R., Yuan Y., Solaymani-Dodaran M., Bazzoli F., Ford A.C. (2018). Global prevalence of, and risk factors for, gastro-oesophageal reflux symptoms: A meta-analysis. Gut.

[6] Thukkani N., Sonnenberg A. (2010). The influence of environmental risk factors in hospitalization for gastro-oesophageal reflux disease-related diagnoses in the United States. Aliment. Pharmacol. Ther..

[7] El-Serag H.B., Petersen N.J., Carter J., Graham D.Y., Richardson P., Genta R.M., Rabeneck L. (2004). Gastroesophageal reflux among different racial groups in the United States. Gastroenterology.

[8] Singh S., Sharma A.N., Murad M.H., Buttar N.S., El-Serag H.B., Katzka D.A., Iyer P.G. (2013). Central adiposity is associated with increased risk of esophageal inflammation, metaplasia, and adenocarcinoma: A systematic review and meta-analysis. Clin. Gastroenterol. Hepatol..

[9] el-Serag H.B., Sonnenberg A. (1998). Opposing time trends of peptic ulcer and reflux disease. Gut.

[10] Fischbach L.A., Nordenstedt H., Kramer J.R., Gandhi S., Dick-Onuoha S., Lewis A., El-Serag H.B. (2012). The association between Barrett’s esophagus and Helicobacter pylori infection: A meta-analysis. Helicobacter.

[11] Rokkas T., Pistiolas D., Sechopoulos P., Robotis I., Margantinis G. (2007). Relationship between Helicobacter pylori infection and esophageal neoplasia: A meta-analysis. Clin. Gastroenterol. Hepatol..

[12] Cameron A.J., Lagergren J., Henriksson C., Nyren O., Locke G.R., Pedersen N.L. (2002). Gastroesophageal reflux disease in monozygotic and dizygotic twins. Gastroenterology.

[13] Mohammed I., Cherkas L.F., Riley S.A., Spector T.D., Trudgill N.J. (2003). Genetic influences in gastro-oesophageal reflux disease: A twin study. Gut.

[14] Naik R.D., Evers L., Vaezi M.F. (2019). Advances in the Diagnosis and Treatment of GERD: New Tricks for an Old Disease. Curr. Treat. Options Gastroenterol..

[15] Chatila A.T., Nguyen M.T.T., Krill T., Roark R., Bilal M., Reep G. (2020). Natural history, pathophysiology and evaluation of gastroesophageal reflux disease. Dis. Mon..

[16] Katz P.O., Dunbar K.B., Schnoll-Sussman F.H., Greer K.B., Yadlapati R., Spechler S.J. (2022). ACG Clinical Guideline for the Diagnosis and Management of Gastroesophageal Reflux Disease. Am. J. Gastroenterol..

[17] Katz P.O., Gerson L.B., Vela M.F. (2013). Guidelines for the diagnosis and management of gastroesophageal reflux disease. Am. J. Gastroenterol..

[18] Eloubeidi M.A., Provenzale D. (1999). Does this patient have Barrett’s esophagus? The utility of predicting Barrett’s esophagus at the index endoscopy. Am. J. Gastroenterol..

[19] Connor M.J., Sharma P. (2004). Chromoendoscopy and magnification endoscopy for diagnosing esophageal cancer and dysplasia. Thorac. Surg. Clin..

[20] Sifrim D., Castell D., Dent J., Kahrilas P.J. (2004). Gastro-oesophageal reflux monitoring: Review and consensus report on detection and definitions of acid, non-acid, and gas reflux. Gut.

[21] Sandhu D.S., Fass R. (2018). Current Trends in the Management of Gastroesophageal Reflux Disease. Gut Liver.

[22] Nabi Z., Reddy D.N. (2016). Endoscopic Management of Gastroesophageal Reflux Disease: Revisited. Clin. Endosc..

[23] Quader F., Gyawali C.P. (2020). 7RECENT advances in endoscopic treatments for gastroesophageal reflux disease. Curr. Treat. Options Gastroenterol..

[24] Lacy B.E., Weiser K., Chertoff J., Fass R., Pandolfino J.E., Richter J.E., Rothstein R.I., Spangler C., Vaezi M.F. (2010). The diagnosis of gastroesophageal reflux disease. Am. J. Med..

[25] Aanen M.C., Weusten B.L., Numans M.E., de Wit N.J., Baron A., Smout A.J. (2006). Diagnostic value of the proton pump inhibitor test for gastro-oesophageal reflux disease in primary care. Aliment. Pharmacol. Ther..

[26] Pritchett J.M., Aslam M., Slaughter J.C., Ness R.M., Garrett C.G., Vaezi M.F. (2009). Efficacy of esophageal impedance/pH monitoring in patients with refractory gastroesophageal reflux disease, on and off therapy. Clin. Gastroenterol. Hepatol..

[27] Lee J.S. (2012). Is Wireless Capsule pH Monitoring Better Than Catheter Systems?. J. Neurogastroenterol. Motil..

[28] Gyawali C.P., Carlson D.A., Chen J.W., Patel A., Wong R.J., Yadlapati R.H. (2020). ACG Clinical Guidelines: Clinical Use of Esophageal Physiologic Testing. Am. J. Gastroenterol..

[29] Neto R.M.L., Herbella F.A.M., Schlottmann F., Patti M.G. (2019). Does DeMeester score still define GERD?. Dis. Esophagus.

[30] Davis T.A., Gyawali C.P. (2024). Refractory Gastroesophageal Reflux Disease: Diagnosis and Management. J. Neurogastroenterol. Motil..

[31] Dhawan I., O’Connell B., Patel A., Schey R., Parkman H.P., Friedenberg F. (2018). Utility of Esophageal High-Resolution Manometry in Clinical Practice: First, Do HRM. Dig. Dis. Sci..

[32] Katzka D.A., Pandolfino J.E., Kahrilas P.J. (2020). Phenotypes of Gastroesophageal Reflux Disease: Where Rome, Lyon, and Montreal Meet. Clin. Gastroenterol. Hepatol..

[33] Dent J., Brun J., Fendrick A., Fennerty M., Janssens J., Kahrilas P., Lauritsen K., Reynolds J., Shaw M., Talley N. (1999). An evidence-based appraisal of reflux disease management—The Genval Workshop Report. Gut.

[34] Quigley E.M., DiBaise J.K. (2001). Non-erosive reflux disease: The real problem in gastro-oesophageal reflux disease. Dig. Liver Dis..

[35] Bond A., Burkitt M.D., Cox T., Smart H.L., Probert C., Haslam N., Sarkar S. (2017). Dual-focus Magnification, High-Definition Endoscopy Improves Pathology Detection in Direct-to-Test Diagnostic Upper Gastrointestinal Endoscopy. J. Gastrointestin Liver Dis..

[36] Qumseya B.J., Wang H., Badie N., Uzomba R.N., Parasa S., White D.L., Wolfsen H., Sharma P., Wallace M.B. (2013). Advanced imaging technologies increase detection of dysplasia and neoplasia in patients with Barrett’s esophagus: A meta-analysis and systematic review. Clin. Gastroenterol. Hepatol..

[37] Song J., Zhang J., Wang J., Guo X., Yu S., Wang J., Liu Y., Dong W. (2015). Meta-analysis of the effects of endoscopy with narrow band imaging in detecting dysplasia in Barrett’s esophagus. Dis. Esophagus.

[38] Borovicka J., Fischer J., Neuweiler J., Netzer P., Gschossmann J., Ehmann T., Bauerfeind P., Dorta G., Zürcher U., Binek J. (2006). Autofluorescence endoscopy in surveillance of Barrett’s esophagus: A multicenter randomized trial on diagnostic efficacy. Endoscopy.

[39] Xiong Y.Q., Ma S.J., Zhou J.H., Zhong X.S., Chen Q. (2016). A meta-analysis of confocal laser endomicroscopy for the detection of neoplasia in patients with Barrett’s esophagus. J. Gastroenterol. Hepatol..

[40] Bhardwaj A., Hollenbeak C.S., Pooran N., Mathew A. (2009). A meta-analysis of the diagnostic accuracy of esophageal capsule endoscopy for Barrett’s esophagus in patients with gastroesophageal reflux disease. Am. J. Gastroenterol..

[41] Sidorenko E.I., Sharma P. (2004). High-resolution chromoendoscopy in the esophagus. Gastrointest. Endosc. Clin. N. Am..

[42] Kiesslich R., Kanzler S., Vieth M., Moehler M., Neidig J., Thanka Nadar B.J., Schilling D., Burg J., Nafe B., Neurath M.F. (2004). Minimal change esophagitis: Prospective comparison of endoscopic and histological markers between patients with non-erosive reflux disease and normal controls using magnifying endoscopy. Dig. Dis..

[43] Mayinger B., Oezturk Y., Stolte M., Faller G., Benninger J., Schwab D., Maiss J., Hahn E.G., Muehldorfer S. (2006). Evaluation of sensitivity and inter- and intra-observer variability in the detection of intestinal metaplasia and dysplasia in Barrett’s esophagus with enhanced magnification endoscopy. Scand. J. Gastroenterol..

[44] Nothmann B.J., Wright J.R., Schuster M.M. (1972). In vivo vital staining as an aid to identification of esophagogastric mucosal junction in man. Am. J. Dig. Dis..

[45] Chaiteerakij R., Rerknimitr R., Kullavanijaya P. (2010). Role of digital chromoendoscopy in detecting minimal change esophageal reflux disease. World J. Gastrointest. Endosc..

[46] Reddymasu S.C., Sharma P. (2008). Advances in endoscopic imaging of the esophagus. Gastroenterol. Clin. N. Am..

[47] Yoshikawa I., Yamasaki M., Yamasaki T., Kume K., Otsuki M. (2005). Lugol chromoendoscopy as a diagnostic tool in so-called endoscopy-negative GERD. Gastrointest. Endosc..

[48] Fock K.M., Teo E.K., Ang T.L., Tan J.Y., Law N.M. (2009). The utility of narrow band imaging in improving the endoscopic diagnosis of gastroesophageal reflux disease. Clin. Gastroenterol. Hepatol..

[49] Kumagai Y., Toi M., Inoue H. (2002). Dynamism of tumour vasculature in the early phase of cancer progression: Outcomes from oesophageal cancer research. Lancet Oncol..

[50] Kumagai Y., Inoue H., Nagai K., Kawano T., Iwai T. (2002). Magnifying endoscopy, stereoscopic microscopy, and the microvascular architecture of superficial esophageal carcinoma. Endoscopy.

[51] Sharma P., Hawes R.H., Bansal A., Gupta N., Curvers W., Rastogi A., Singh M., Hall M., Mathur S.C., Wani S.B. (2013). Standard endoscopy with random biopsies versus narrow band imaging targeted biopsies in Barrett’s oesophagus: A prospective, international, randomised controlled trial. Gut.

[52] Mann R., Gajendran M., Perisetti A., Goyal H., Saligram S., Umapathy C. (2021). Advanced Endoscopic Imaging and Interventions in GERD: An Update and Future Directions. Front. Med..

[53] Pohl J., Lotterer E., Balzer C., Sackmann M., Schmidt K.D., Gossner L., Schaab C., Frieling T., Medve M., Mayer G. (2009). Computed virtual chromoendoscopy versus standard colonoscopy with targeted indigocarmine chromoscopy: A randomised multicentre trial. Gut.

[54] Kim M.S., Choi S.R., Roh M.H., Lee J.H., Jang J.S., Kim B.G., Kim S.O., Han J.S., Hsing C.T. (2011). Efficacy of I-scan endoscopy in the diagnosis of gastroesophageal reflux disease with minimal change. Clin. Endosc..

[55] Netinatsunton N., Sottisuporn J., Attasaranya S., Witeerungrot T., Chamroonkul N., Jongboonyanuparp T., Geater A., Ovartlarnporn B. (2016). i-Scan detection of minimal change esophagitis in dyspeptic patients with or without Gastroesophageal Reflux disease. BMC Gastroenterol..

[56] Filip M., Iordache S., Săftoiu A., Ciurea T. (2011). Autofluorescence imaging and magnification endoscopy. World J. Gastroenterol..

[57] East J.E., Vleugels J.L., Roelandt P., Bhandari P., Bisschops R., Dekker E., Hassan C., Horgan G., Kiesslich R., Longcroft-Wheaton G. (2016). Advanced endoscopic imaging: European Society of Gastrointestinal Endoscopy (ESGE) Technology Review. Endoscopy.

[58] Kara M.A., Peters F.P., Ten Kate F.J., Van Deventer S.J., Fockens P., Bergman J.J. (2005). Endoscopic video autofluorescence imaging may improve the detection of early neoplasia in patients with Barrett’s esophagus. Gastrointest. Endosc..

[59] Boerwinkel D.F., Holz J.A., Aalders M.C., Visser M., Meijer S.L., Van Berge Henegouwen M.I., Weusten B.L., Bergman J.J. (2014). Third-generation autofluorescence endoscopy for the detection of early neoplasia in Barrett’s esophagus: A pilot study. Dis. Esophagus.

[60] Wani S., Sharma P. (2007). Endoscopic surface imaging of Barrett’s Esophagus: An optimistic view. Gastroenterology.

[61] Sharma P., Topalovski M., Mayo M.S., Weston A.P. (2001). Methylene blue chromoendoscopy for detection of short-segment Barrett’s esophagus. Gastrointest. Endosc..

[62] Kiesslich R., Gossner L., Goetz M., Dahlmann A., Vieth M., Stolte M., Hoffman A., Jung M., Nafe B., Galle P.R. (2006). In vivo histology of Barrett’s esophagus and associated neoplasia by confocal laser endomicroscopy. Clin. Gastroenterol. Hepatol..

[63] Becker V., Vieth M., Bajbouj M., Schmid R.M., Meining A. (2008). Confocal laser scanning fluorescence microscopy for in vivo determination of microvessel density in Barrett’s esophagus. Endoscopy.

[64] Sharma V.K. (2014). Role of endoscopy in GERD. Gastroenterol. Clin. N. Am..

[65] Park J., Cho Y.K., Kim J.H. (2018). Current and Future Use of Esophageal Capsule Endoscopy. Clin. Endosc..

[66] Galmiche J.P., Sacher-Huvelin S., Coron E., Cholet F., Soussan E.B., Sébille V., Filoche B., d’Abrigeon G., Antonietti M., Robaszkiewicz M. (2008). Screening for esophagitis and Barrett’s esophagus with wireless esophageal capsule endoscopy: A multicenter prospective trial in patients with reflux symptoms. Am. J. Gastroenterol..

[67] Odze R.D., Goldblum J., Kaul V. (2021). Role of Wide-Area Transepithelial Sampling With 3D Computer-Assisted Analysis in the Diagnosis and Management of Barrett’s Esophagus. Clin. Transl. Gastroenterol..

[68] Smith M.S., Ikonomi E., Bhuta R., Iorio N., Kataria R.D., Kaul V., Gross S.A. (2019). Wide-area transepithelial sampling with computer-assisted 3-dimensional analysis (WATS) markedly improves detection of esophageal dysplasia and Barrett’s esophagus: Analysis from a prospective multicenter community-based study. Dis. Esophagus.

[69] Shaheen N.J., Falk G.W., Iyer P.G., Souza R.F., Yadlapati R.H., Sauer B.G., Wani S. (2022). Diagnosis and Management of Barrett’s Esophagus: An Updated ACG Guideline. Am. J. Gastroenterol..

[70] Krishna Chandar A., Sharma A., Chak A. (2020). Novel Screening Alternatives for Barrett Esophagus. Gastroenterol. Hepatol..

[71] Pohl H., Welch H.G. (2005). The role of overdiagnosis and reclassification in the marked increase of esophageal adenocarcinoma incidence. J. Natl. Cancer Inst..

[72] Thrift A.P. (2016). The epidemic of oesophageal carcinoma: Where are we now?. Cancer Epidemiol..

[73] Shaheen N.J., Falk G.W., Iyer P.G., Gerson L.B. (2016). ACG Clinical Guideline: Diagnosis and Management of Barrett’s Esophagus. Am. J. Gastroenterol..

[74] Spechler S.J., Sharma P., Souza R.F., Inadomi J.M., Shaheen N.J. (2011). American Gastroenterological Association medical position statement on the management of Barrett’s esophagus. Gastroenterology.

[75] Qumseya B., Sultan S., Bain P., Jamil L., Jacobson B., Anandasabapathy S., Agrawal D., Buxbaum J.L., Fishman D.S., Gurudu S.R. (2019). ASGE guideline on screening and surveillance of Barrett’s esophagus. Gastrointest. Endosc..

[76] Muthusamy V.R., Wani S., Gyawali C.P., Komanduri S. (2022). AGA Clinical Practice Update on New Technology and Innovation for Surveillance and Screening in Barrett’s Esophagus: Expert Review. Clin. Gastroenterol. Hepatol..

[77] Sharma P., Falk G.W., Weston A.P., Reker D., Johnston M., Sampliner R.E. (2006). Dysplasia and cancer in a large multicenter cohort of patients with Barrett’s esophagus. Clin. Gastroenterol. Hepatol..

[78] Evans J.A., Early D.S., Fukami N., Ben-Menachem T., Chandrasekhara V., Chathadi K.V., Decker G.A., Fanelli R.D., Fisher D.A., Foley K.Q. (2012). The role of endoscopy in Barrett’s esophagus and other premalignant conditions of the esophagus. Gastrointest. Endosc..

[79] Dulai G.S., Guha S., Kahn K.L., Gornbein J., Weinstein W.M. (2002). Preoperative prevalence of Barrett’s esophagus in esophageal adenocarcinoma: A systematic review. Gastroenterology.

[80] Verbeek R.E., Leenders M., Ten Kate F.J., van Hillegersberg R., Vleggaar F.P., van Baal J.W., van Oijen M.G., Siersema P.D. (2014). Surveillance of Barrett’s esophagus and mortality from esophageal adenocarcinoma: A population-based cohort study. Am. J. Gastroenterol..

[81] Iyer P.G., Chak A. (2016). Can endosheath technology open primary care doors to Barrett’s esophagus screening by transnasal endoscopy?. Endoscopy.

[82] Wilkinson J., Wade A., Thomas S.J., Jenner B., Hodgkinson V., Coyle C. (2019). Randomized clinical trial: A double-blind, placebo-controlled study to assess the clinical efficacy and safety of alginate-antacid (Gaviscon Double Action) chewable tablets in patients with gastro-oesophageal reflux disease. Eur. J. Gastroenterol. Hepatol..

[83] Young A., Kumar M.A., Thota P.N. (2020). GERD: A practical approach. Cleve Clin. J. Med..

[84] Laine L., DeVault K., Katz P., Mitev S., Lowe J., Hunt B., Spechler S. (2023). Vonoprazan Versus Lansoprazole for Healing and Maintenance of Healing of Erosive Esophagitis: A Randomized Trial. Gastroenterology.

[85] Kaltenbach T., Crockett S., Gerson L.B. (2006). Are lifestyle measures effective in patients with gastroesophageal reflux disease? An evidence-based approach. Arch. Intern. Med..

[86] El-Serag H.B., Satia J.A., Rabeneck L. (2005). Dietary intake and the risk of gastro-oesophageal reflux disease: A cross sectional study in volunteers. Gut.

[87] Newberry C., Lynch K. (2019). The role of diet in the development and management of gastroesophageal reflux disease: Why we feel the burn. J. Thorac. Dis..

[88] Ness-Jensen E., Lindam A., Lagergren J., Hveem K. (2014). Tobacco smoking cessation and improved gastroesophageal reflux: A prospective population-based cohort study: The HUNT study. Am. J. Gastroenterol..

[89] Kohata Y., Fujiwara Y., Watanabe T., Kobayashi M., Takemoto Y., Kamata N., Yamagami H., Tanigawa T., Shiba M., Watanabe T. (2016). Long-Term Benefits of Smoking Cessation on Gastroesophageal Reflux Disease and Health-Related Quality of Life. PLoS ONE.

[90] Hinder R.A., Filipi C.J., Wetscher G., Neary P., DeMeester T.R., Perdikis G. (1994). Laparoscopic Nissen fundoplication is an effective treatment for gastroesophageal reflux disease. Ann. Surg..

[91] Katzka D.A., Kahrilas P.J. (2020). Advances in the diagnosis and management of gastroesophageal reflux disease. BMJ.

[92] Banerjee R., Pratap N., Kalpala R., Reddy D.N. (2014). Effect of electrical stimulation of the lower esophageal sphincter using endoscopically implanted temporary stimulation leads in patients with reflux disease. Surg. Endosc..

[93] Sumi K., Inoue H., Kobayashi Y., Iwaya Y., Abad M.R.A., Fujiyoshi Y., Shimamura Y., Ikeda H., Onimaru M. (2021). Endoscopic treatment of proton pump inhibitor-refractory gastroesophageal reflux disease with anti-reflux mucosectomy: Experience of 109 cases. Dig. Endosc..

[94] Fass R., Cahn F., Scotti D.J., Gregory D.A. (2017). Systematic review and meta-analysis of controlled and prospective cohort efficacy studies of endoscopic radiofrequency for treatment of gastroesophageal reflux disease. Surg. Endosc..

[95] McCarty T.R., Itidiare M., Njei B., Rustagi T. (2018). Efficacy of transoral incisionless fundoplication for refractory gastroesophageal reflux disease: A systematic review and meta-analysis. Endoscopy.

[96] Zacherl J., Roy-Shapira A., Bonavina L., Bapaye A., Kiesslich R., Schoppmann S.F., Kessler W.R., Selzer D.J., Broderick R.C., Lehman G.A. (2015). Endoscopic anterior fundoplication with the Medigus Ultrasonic Surgical Endostapler (MUSE™) for gastroesophageal reflux disease: 6-month results from a multi-center prospective trial. Surg. Endosc..

[97] Hu H.Q., Li H.K., Xiong Y., Zhang X.B., Zhi J.L., Wang X.X., Ling-Hu E.Q. (2018). Peroral endoscopic cardial constriction in gastroesophageal reflux disease. Medicine.

[98] Schiefke I., Neumann S., Zabel-Langhennig A., Moessner J., Caca K. (2005). Use of an endoscopic suturing device (the “ESD”) to treat patients with gastroesophageal reflux disease, after unsuccessful EndoCinch endoluminal gastroplication: Another failure. Endoscopy.

[99] Schwartz M.P., Wellink H., Gooszen H.G., Conchillo J.M., Samsom M., Smout A.J. (2007). Endoscopic gastroplication for the treatment of gastro-oesophageal reflux disease: A randomised, sham-controlled trial. Gut.

[100] Rothstein R., Filipi C., Caca K., Pruitt R., Mergener K., Torquati A., Haber G., Chen Y., Chang K., Wong D. (2006). Endoscopic full-thickness plication for the treatment of gastroesophageal reflux disease: A randomized, sham-controlled trial. Gastroenterology.

[101] Ramage J.I., Rothstein R.I., Edmundowicz S.A., Chen Y.K., Lehman G.A., Fennerty M.B., Sharma V.K., Carr-Locke D.L., Gostout C.J. (2006). Endoscopically Placed Titanium Plicator for GERD: Pivotal Phase—Preliminary 6-Month Results. Gastrointest. Endosc..

[102] Sud R., Puri R., Chung S., Cotton P.B., Christopher G.J., Hawes R.H., Kalloo A.N., Kantsevoy S., Pasricha P.J. (2006). The His-Wiz antireflux procedure results in symptomatic and pH improvement at 1 year of follow-up. Gastrointest. Endosc..

[103] Seleem W.M., Hanafy A.S., Mohamed S.I. (2018). Endoscopic management of refractory gastroesophageal reflux disease. Scand. J. Gastroenterol..

[104] Benias P.C., D’Souza L., Lan G., Gluckman C., Inamdar S., Trindade A.J., Miller L.S., Carr-Locke D.L. (2018). Initial experience with a novel resection and plication (RAP) method for acid reflux: A pilot study. Endosc. Int. Open.

[105] Rodríguez L., Rodriguez P.A., Gómez B., Netto M.G., Crowell M.D., Soffer E. (2016). Electrical stimulation therapy of the lower esophageal sphincter is successful in treating GERD: Long-term 3-year results. Surg. Endosc..

[106] Paireder M., Kristo I., Asari R., Jomrich G., Steindl J., Rieder E., Schoppmann S.F. (2021). Effect of electrical stimulation therapy of the lower esophageal sphincter in GERD patients with ineffective esophageal motility. Surg. Endosc..

[107] Nabi Z., Reddy D.N. (2019). Update on Endoscopic Approaches for the Management of Gastroesophageal Reflux Disease. Gastroenterol. Hepatol..

[108] Inoue H., Ito H., Ikeda H., Sato C., Sato H., Phalanusitthepha C., Hayee B., Eleftheriadis N., Kudo S.E. (2014). Anti-reflux mucosectomy for gastroesophageal reflux disease in the absence of hiatus hernia: A pilot study. Ann. Gastroenterol..

[109] Garg R., Mohammed A., Singh A., Schleicher M., Thota P.N., Rustagi T., Sanaka M.R. (2022). Anti-reflux mucosectomy for refractory gastroesophageal reflux disease: A systematic review and meta-analysis. Endosc. Int. Open.

[110] Auyang E.D., Carter P., Rauth T., Fanelli R.D. (2013). SAGES clinical spotlight review: Endoluminal treatments for gastroesophageal reflux disease (GERD). Surg. Endosc..

[111] Triadafilopoulos G. (2014). Stretta: A valuable endoscopic treatment modality for gastroesophageal reflux disease. World J. Gastroenterol..

[112] Funk L.M., Zhang J.Y., Drosdeck J.M., Melvin W.S., Walker J.P., Perry K.A. (2015). Long-term cost-effectiveness of medical, endoscopic and surgical management of gastroesophageal reflux disease. Surgery.

[113] Yan C., Liang W.T., Wang Z.G., Hu Z.W., Wu J.M., Zhang C., Chen M.P. (2015). Comparison of Stretta procedure and toupet fundoplication for gastroesophageal reflux disease-related extra-esophageal symptoms. World J. Gastroenterol..

[114] Liang W.T., Yan C., Wang Z.G., Wu J.M., Hu Z.W., Zhan X.L., Wang F., Ma S.S., Chen M.P. (2015). Early and Midterm Outcome After Laparoscopic Fundoplication and a Minimally Invasive Endoscopic Procedure in Patients with Gastroesophageal Reflux Disease: A Prospective Observational Study. J. Laparoendosc. Adv. Surg. Tech. A.

[115] Dughera L., Rotondano G., De Cento M., Cassolino P., Cisarò F. (2014). Durability of Stretta Radiofrequency Treatment for GERD: Results of an 8-Year Follow-Up. Gastroenterol. Res. Pract..

[116] Corley D.A., Katz P., Wo J.M., Stefan A., Patti M., Rothstein R., Edmundowicz S., Kline M., Mason R., Wolfe M.M. (2003). Improvement of gastroesophageal reflux symptoms after radiofrequency energy: A randomized, sham-controlled trial. Gastroenterology.

[117] Arts J., Bisschops R., Blondeau K., Farré R., Vos R., Holvoet L., Caenepeel P., Lerut A., Tack J. (2012). A double-blind sham-controlled study of the effect of radiofrequency energy on symptoms and distensibility of the gastro-esophageal junction in GERD. Am. J. Gastroenterol..

[118] Malcangio M., Bowery N.G. (1993). GABAB receptor-mediated inhibition of forskolin-stimulated cyclic AMP accumulation in rat spinal cord. Neurosci. Lett..

[119] Reymunde A., Santiago N. (2007). Long-term results of radiofrequency energy delivery for the treatment of GERD: Sustained improvements in symptoms, quality of life, and drug use at 4-year follow-up. Gastrointest. Endosc..

[120] Dughera L., Navino M., Cassolino P., De Cento M., Cacciotella L., Cisarò F., Chiaverina M. (2011). Long-Term Results of Radiofrequency Energy Delivery for the Treatment of GERD: Results of a Prospective 48-Month Study. Diagn. Ther. Endosc..

[121] Noar M.D., Lotfi-Emran S. (2007). Sustained improvement in symptoms of GERD and antisecretory drug use: 4-year follow-up of the Stretta procedure. Gastrointest. Endosc..

[122] Mattar S.G., Qureshi F., Taylor D., Schauer P.R. (2006). Treatment of refractory gastroesophageal reflux disease with radiofrequency energy (Stretta) in patients after Roux-en-Y gastric bypass. Surg. Endosc..

[123] Noar M., Squires P., Noar E., Lee M. (2014). Long-term maintenance effect of radiofrequency energy delivery for refractory GERD: A decade later. Surg. Endosc..

[124] Testoni P.A., Mazzoleni G., Testoni S.G. (2016). Transoral incisionless fundoplication for gastro-esophageal reflux disease: Techniques and outcomes. World J. Gastrointest. Pharmacol. Ther..

[125] Fass R. (2017). An Overview of Transoral Incisionless Fundoplication and Magnetic Sphincter Augmentation for GERD. Gastroenterol. Hepatol..

[126] Chang K.J., Bell R. (2020). Transoral Incisionless Fundoplication. Gastrointest. Endosc. Clin. N. Am..

[127] Trad K.S., Barnes W.E., Simoni G., Shughoury A.B., Mavrelis P.G., Raza M., Heise J.A., Turgeon D.G., Fox M.A. (2015). Transoral incisionless fundoplication effective in eliminating GERD symptoms in partial responders to proton pump inhibitor therapy at 6 months: The TEMPO Randomized Clinical Trial. Surg. Innov..

[128] Witteman B.P., Strijkers R., de Vries E., Toemen L., Conchillo J.M., Hameeteman W., Dagnelie P.C., Koek G.H., Bouvy N.D. (2012). Transoral incisionless fundoplication for treatment of gastroesophageal reflux disease in clinical practice. Surg. Endosc..

[129] Hunter J.G., Kahrilas P.J., Bell R.C., Wilson E.B., Trad K.S., Dolan J.P., Perry K.A., Oelschlager B.K., Soper N.J., Snyder B.E. (2015). Efficacy of transoral fundoplication vs omeprazole for treatment of regurgitation in a randomized controlled trial. Gastroenterology.

[130] Trad K.S., Barnes W.E., Prevou E.R., Simoni G., Steffen J.A., Shughoury A.B., Raza M., Heise J.A., Fox M.A., Mavrelis P.G. (2018). The TEMPO Trial at 5 Years: Transoral Fundoplication (TIF 2.0) Is Safe, Durable, and Cost-effective. Surg. Innov..

[131] Huang X., Chen S., Zhao H., Zeng X., Lian J., Tseng Y., Chen J. (2017). Efficacy of transoral incisionless fundoplication (TIF) for the treatment of GERD: A systematic review with meta-analysis. Surg. Endosc..

[132] Testoni P.A., Testoni S., Mazzoleni G., Vailati C., Passaretti S. (2015). Long-term efficacy of transoral incisionless fundoplication with Esophyx (Tif 2.0) and factors affecting outcomes in GERD patients followed for up to 6 years: A prospective single-center study. Surg. Endosc..

[133] Pleskow D., Rothstein R., Lo S., Hawes R., Kozarek R., Haber G., Gostout C., Lembo A. (2005). Endoscopic full-thickness plication for the treatment of GERD: 12-month follow-up for the North American open-label trial. Gastrointest. Endosc..

[134] Weitzendorfer M., Spaun G.O., Antoniou S.A., Witzel K., Emmanuel K., Koch O.O. (2018). Clinical feasibility of a new full-thickness endoscopic plication device (GERDx™) for patients with GERD: Results of a prospective trial. Surg. Endosc..

[135] Kalapala R., Karyampudi A., Nabi Z., Darisetty S., Jagtap N., Ramchandani M., Gupta R., Lakhtakia S., Goud R., Venkat Rao G. (2022). Endoscopic full-thickness plication for the treatment of PPI-dependent GERD: Results from a randomised, sham controlled trial. Gut.

[136] Schilling D., Kiesslich R., Galle P.R., Riemann J.F. (2005). Endoluminal therapy of GERD with a new endoscopic suturing device. Gastrointest. Endosc..

[137] Schwartz M.P., Smout A.J. (2007). Review article: The endoscopic treatment of gastro-oesophageal reflux disease. Aliment. Pharmacol. Ther..

[138] Ozawa S., Kumai K., Higuchi K., Arakawa T., Kato M., Asaka M., Katada N., Kuwano H., Kitajima M. (2009). Short-term and long-term outcome of endoluminal gastroplication for the treatment of GERD: The first multicenter trial in Japan. J. Gastroenterol..

[139] Yew K.C., Chuah S.K. (2013). Antireflux endoluminal therapies: Past and present. Gastroenterol. Res. Pract..

[140] Mosler P., Aziz A.M., Hieston K., Filipi C., Lehman G. (2008). Evaluation of supplemental cautery during endoluminal gastroplication for the treatment of gastroesophageal reflux disease. Surg. Endosc..

[141] Mahmood Z., Byrne P.J., McMahon B.P., Murphy E.M., Arfin Q., Ravi N., Weir D.G., Reynolds J.V. (2006). Comparison of transesophageal endoscopic plication (TEP) with laparoscopic Nissen fundoplication (LNF) in the treatment of uncomplicated reflux disease. Am. J. Gastroenterol..

[142] Chuttani R., Sud R., Sachdev G., Puri R., Kozarek R., Haber G., Pleskow D., Zaman M., Lembo A. (2003). A novel endoscopic full-thickness plicator for the treatment of GERD: A pilot study. Gastrointest. Endosc..

[143] Pleskow D., Rothstein R., Kozarek R., Haber G., Gostout C., Lo S., Hawes R., Lembo A. (2008). Endoscopic full-thickness plication for the treatment of GERD: Five-year long-term multicenter results. Surg. Endosc..

[144] Jeansonne L.O., White B.C., Nguyen V., Jafri S.M., Swafford V., Katchooi M., Khaitan L., Davis S.S., Smith C.D., Sedghi S. (2009). Endoluminal full-thickness plication and radiofrequency treatments for GERD: An outcomes comparison. Arch. Surg..

[145] Cohen L.B., Johnson D.A., Ganz R.A., Aisenberg J., Devière J., Foley T.R., Haber G.B., Peters J.H., Lehman G.A. (2005). Enteryx implantation for GERD: Expanded multicenter trial results and interim postapproval follow-up to 24 months. Gastrointest. Endosc..

[146] Buquet C., Desmidt A., Charlier J., Querleu D. (1992). Evaluation of spatial discrimination performances of newborn infants with the visual pursuit of structured stimuli. C. R. Acad. Sci. III.

[147] Fockens P., Bruno M.J., Gabbrielli A., Odegaard S., Hatlebakk J., Allescher H.D., Rösch T., Rhodes M., Bastid C., Rey J. (2004). Endoscopic augmentation of the lower esophageal sphincter for the treatment of gastroesophageal reflux disease: Multicenter study of the Gatekeeper Reflux Repair System. Endoscopy.

[148] Fockens P., Cohen L., Edmundowicz S.A., Binmoeller K., Rothstein R.I., Smith D., Lin E., Nickl N., Overholt B., Kahrilas P.J. (2010). Prospective randomized controlled trial of an injectable esophageal prosthesis versus a sham procedure for endoscopic treatment of gastroesophageal reflux disease. Surg. Endosc..

[149] Lo W.K., Mashimo H. (2015). Critical Assessment of Endoscopic Techniques for Gastroesophageal Reflux Disease. J. Clin. Gastroenterol..

[150] Ganz R.A., Fallon E., Wittchow T., Klein D. (2009). A new injectable agent for the treatment of GERD: Results of the Durasphere pilot trial. Gastrointest. Endosc..

[151] Rouphael C., Padival R., Sanaka M.R., Thota P.N. (2018). Endoscopic Treatments of GERD. Curr. Treat. Options Gastroenterol..

[152] Feretis C., Benakis P., Dimopoulos C., Dailianas A., Filalithis P., Stamou K.M., Manouras A., Apostolidis N. (2001). Endoscopic implantation of Plexiglas (PMMA) microspheres for the treatment of GERD. Gastrointest. Endosc..

